# The *Xanthomonas campestris* Type III Effector XopJ Targets the Host Cell Proteasome to Suppress Salicylic-Acid Mediated Plant Defence

**DOI:** 10.1371/journal.ppat.1003427

**Published:** 2013-06-13

**Authors:** Suayib Üstün, Verena Bartetzko, Frederik Börnke

**Affiliations:** Friedrich-Alexander-University Erlangen-Nuremberg, Department of Biology, Division of Biochemistry, Erlangen, Germany; Michigan State University, United States of America

## Abstract

The phytopathogenic bacterium *Xanthomonas campestris* pv. *vesicatoria* (Xcv) requires type III effector proteins (T3Es) for virulence. After translocation into the host cell, T3Es are thought to interact with components of host immunity to suppress defence responses. XopJ is a T3E protein from Xcv that interferes with plant immune responses; however, its host cellular target is unknown. Here we show that XopJ interacts with the proteasomal subunit RPT6 in yeast and *in planta* to inhibit proteasome activity. A C235A mutation within the catalytic triad of XopJ as well as a G2A exchange within the N-terminal myristoylation motif abolishes the ability of XopJ to inhibit the proteasome. Xcv Δ*xopJ* mutants are impaired in growth and display accelerated symptom development including tissue necrosis on susceptible pepper leaves. Application of the proteasome inhibitor MG132 restored the ability of the Xcv Δ*xopJ* to attenuate the development of leaf necrosis. The XopJ dependent delay of tissue degeneration correlates with reduced levels of salicylic acid (SA) and changes in defence- and senescence-associated gene expression. Necrosis upon infection with Xcv Δ*xopJ* was greatly reduced in pepper plants with reduced expression of *NPR1*, a central regulator of SA responses, demonstrating the involvement of SA-signalling in the development of XopJ dependent phenotypes. Our results suggest that XopJ-mediated inhibition of the proteasome interferes with SA-dependent defence response to attenuate onset of necrosis and to alter host transcription. A central role of the proteasome in plant defence is discussed.

## Author Summary

Many bacteria that are pathogens for mammals, insects or plants use a specialized apparatus called the type III secretion system to inject a diverse set of effector proteins into the cytoplasm of their eukaryotic host cells in order to alter cellular processes in favour of the pathogen's lifestyle. However, direct cellular targets have been identified for only a few effector proteins and the elucidation of their mode of action is of fundamental interest for the understanding of bacterial virulence strategies. The effector XopJ from the phytopathogen *Xanthomonas campestris* pv. *vesicatoria* (Xcv), the causal agent of bacterial spot disease on tomato and pepper plants, belongs to the YopJ-superfamily of effector proteins. Members of this family are found among plant and animal pathogens, as well as plant symbionts. We show here that within plant cells XopJ targets the proteasomal subunit RPT6 to suppress host proteasome activity and thus protein turnover. In pepper leaves, this leads to reduced accumulation of the defence hormone salicylic acid (SA) and also attenuates SA-mediated defence responses such as tissue degeneration and defence gene expression. XopJ from Xcv is the first example of a bacterial effector protein targeting the host proteasome and our results also suggest a central role of the proteasome in plant immunity.

## Introduction

Plants have to protect themselves from a plethora of microbial enemies. In a first layer of defence, conserved microbial molecules called PAMPs/MAMPs (pathogen/microbe-associated molecular patterns) are recognized on the cell surface which then leads to the induction of a number of defence responses, including the generation of reactive oxygen species, the initiation of MAP kinase signalling, *PR*-gene expression, and callose depositions at the cell wall [1]. Collectively, these responses are sufficient to prevent multiplication and spread of a broad range of potential pathogens and mostly result in PTI (PAMP-triggered immunity). To overcome this barrier many gram-negative plant pathogenic bacteria have acquired a highly conserved type III secretion system (T3SS) which enables them to inject so called type III effector proteins (T3Es) into the plant cell. These T3Es are targeted to a number of cellular compartments where they influence host cellular processes to promote pathogen multiplication and disease [2], [3]. Many T3Es are enzymes (e.g. phosphotransferases, phospholyases, proteases, E3 ligases, and acetyltransferases), while others have no obvious enzymatic activity or act as transcription factors. The picture that emerges from research in the past few years is that the majority of T3Es acts to suppress basal defence responses and innate immunity by interfering with e.g. defence signal transduction, vesicle trafficking, gene expression, and RNA metabolism [2]–[5]. However, the exact mechanism by which they accomplish their function remains unknown for most of T3Es identified to date. In response to defence suppression by T3Es, plants have acquired the ability to recognize specific effector proteins through resistance (R) proteins. In this second layer of defence effector recognition results in an effective immune response which is often accompanied by rapid, localized cell death, termed the hypersensitive response (HR), eventually restricting bacterial spread and leading to effector triggered immunity (ETI) [6].

One of the most diverse and widely distributed families of T3Es is the YopJ family of cysteine proteases/acetyltransferases [7]–[9]. Members of this large family of T3Es are found among both plant and animal pathogens as well as plant symbionts and a characteristic feature of these proteins is their catalytic triad consisting of the amino acids histidine, glutamic/aspartic acid, and a cysteine. YopJ from *Yersinia pestis*, the archetypal member of this effector family, has been shown to possess acetyltransferase activity. During infection of mammalian cells YopJ inhibits MAP kinase signalling by acetylating a serine or threonine within the activation loop of MKK6, preventing the phosphorylation of theses residues and thereby blocking signal transduction [10]. However, other experiments have demonstrated de-sumoylating and de-ubiquitinating activity for YopJ [11]–[13] although direct targets of these protease activities remain to be determined. More than 10 YopJ homologues have been identified in plant pathogenic bacteria including *Pseudomonas* (HopZ-family), *Ralstonia* (PopP1 and PopP2), *Erwinia* (ORFB), and *Xanthomonas* (AvrRxv, AvrXv4, AvrBsT and XopJ) species as well as the plant symbiont *Rhizobium* (Y4LO) [7]–[9]. HopZ1a from *P. syringae* has recently been shown to target GmHID1 (2- hydroxyisoflavone dehydratase), an enzyme involved in the biosynthesis of isoflavones in soybean. The interaction between the effector protein and GmHID1 leads to the degradation of the enzyme which eventually suppresses the synthesis of the defence compound diazedin and leads to enhanced bacterial multiplication [14]. The mechanism by which HopZ1a causes degradation of GmHID1 is currently unknown. Although it requires an intact catalytic triad, no biochemical activity of HopZ1a could be demonstrated. In another study, Lee et al. [15] could recently show that HopZ1a possess acetyltransferase activity that is activated by the eukaryotic factor phytic acid (inositolhexakisphosphate). HopZ1a was able to acetylate itself and tubulin. In plant cells, HopZ1a causes a dramatic decrease in microtubule networks, disrupts the plant secretory pathway and suppresses cell wall-mediated defense [15]. Another T3E with demonstrated acetyltransferase activity is PopP2 from *Ralstonia solanacearum* [16]. The effector autoacetylates on a particular lysine residue that is conserved among all members of the YopJ family and has also been described as being required for HopZ1a acetyltransferase activity [15]. Although PopP2's acetyltransferase activity is required for proper recognition by the cognate RRS1-R R protein its acetylation target during virulence is currently unknown [16].

XopJ is one of the YopJ-family members present in a number of *Xanthomonas campestris* pv. *vesicatoria* (*Xcv*) strains [17], [18]. XopJ is attached to the plasma membrane of plant cells through a myristoylation motif and has been shown to block protein secretion [19], [20]. Moreover, transgenic expression of XopJ in *Arabidopsis* suppressed callose deposition elicited by an avirulent bacteria indicating that the effector interferes with cell wall – associated defense responses [19]. Mutants with an alanine replacement of the catalytic cysteine residue (C235A) are abrogated in the virulence function of XopJ, indicating that the cellular functions of XopJ are accomplished through its enzymatic activity [19]. However, neither the cellular target(s) nor the biochemical activity of XopJ has been determined so far.

Aim of the present study was to gain insights into the molecular function of XopJ through the identification of potential target proteins. Our results show that XopJ interacts with RPT6 a subunit of the 19S regulatory particle within the 26S proteasome to inhibit proteasome activity. This prevents accumulation of the defence phytohormone salicylic acid (SA) and attenuates SA mediated symptom development as well as pathogen-induced senescence.

## Results

### Identification of XopJ interacting proteins

**Figure 1:**
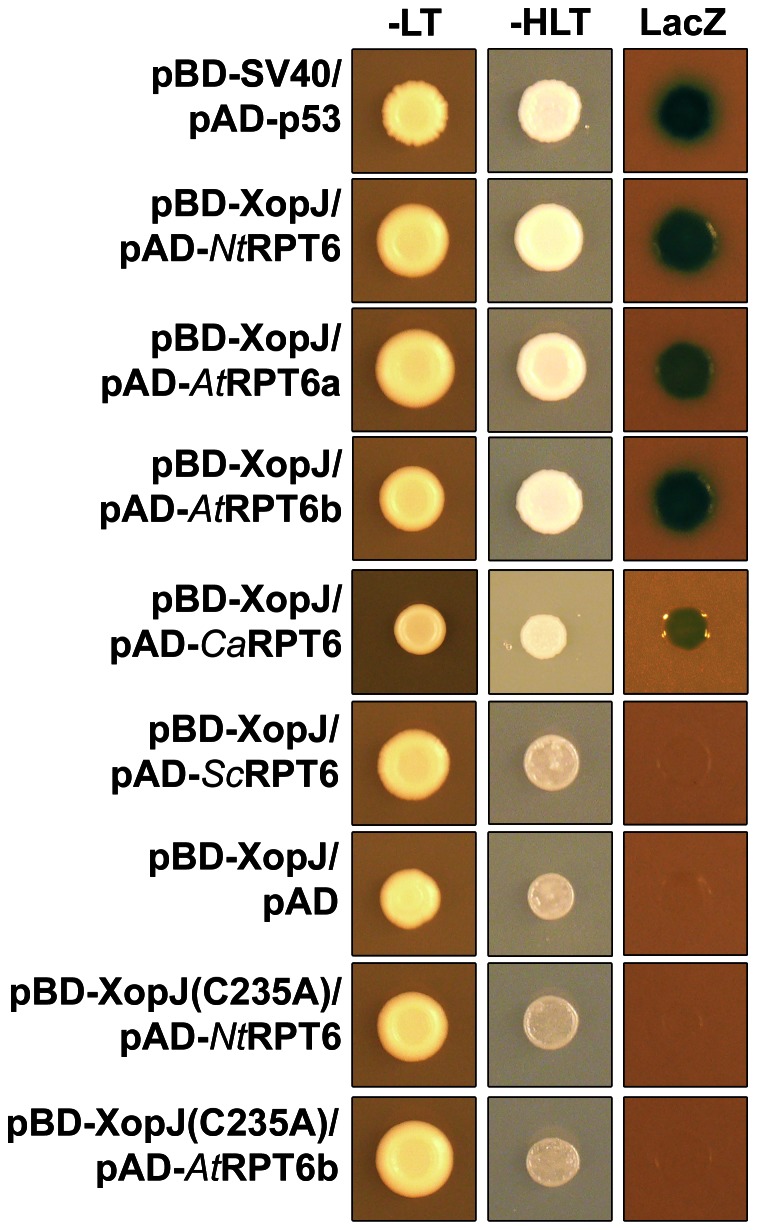
Interaction of XopJ with RPT6 in Yeast Two-Hybrid Assays. XopJ fused to the GAL4 DNA binding domain (BD) was expressed in combination with RPT6 fused to the GAL4 activation domain (AD) in yeast strain Y190. Cells were grown on selective media before a LacZ filter assay was performed. pSV40/p53 served as positive control while the empty AD vector served as negative control. NtRPT6, *N. tabacum* RPT6; AtRPT6a, *A. thaliana* RPT6 isoform a; AtRPT6b, *A. thaliana* RPT6 isoform b; ScRPT6, *S. cerevisiae* RPT6. – LT, yeast growth on medium without Leu and Trp. – HLT, yeast growth on medium lacking His, Leu, and Trp, indicating expression of the *HIS3* reporter gene. LacZ, activity of the *lacZ* reporter gene.

In order to identify potential XopJ target proteins in plant cells, yeast two-hybrid screens were conducted with XopJ as a bait and an *Arabidopsis* and tobacco cDNA library, respectively, as a prey. Neither *Arabidopsis* nor tobacco is a host plant for *Xcv*; however, we have previously established that XopJ inhibits basal defence responses in both species [19]. Therefore, it appears highly likely that XopJ targets are conserved in different plants irrespective of their susceptibility towards *Xcv*. One protein identified as an interaction partner of XopJ in both libraries was RPT6 (Figure 1). RPT6 (regulatory particle ATPase 6) is one of six ATPases of the 19S regulatory particle of the 26S proteasome involved in the degradation of ubiquitinated substrates [21]. The six RPT subunits form a ring that consumes ATP to facilitate substrate unfolding and channel opening, which is required for translocation of substrates from the 19S regulatory particle into the proteolytic 20S core particle of the proteasome [22]. The Arabidopsis genome encodes two RPT6 genes (RPT6a, At5g19990 and RPT6b, At5g20000) which are directly arranged in tandem and expression of both genes is supported by cDNAs (www.arabidopsis.org). Both protein sequences share 97% identity with each other (Figure S1); however, screening the Arabidopsis two-hybrid library recovered only RPT6a as an in XopJ interacting protein and thus the ability of RPT6b to bind XopJ was investigated in a direct interaction assay in yeast. As shown in Figure 1 pair wise transformation of BD-XopJ and AD-AtRPT6b did also result in activation of reporter genes indicating that XopJ/RPT6 interaction is not specific for AtRPT6a. The RPT6 protein sequence is highly conserved across phylogeny with 81 to 82% identity between Arabidopsis RPT6a and RPT6 from human and 70% identity between the RPT6 sequences from Arabidopsis and yeast (Figure S1). In order to assess whether XopJ could interact with RPT6 from another phylum, we tested its interaction with RPT6 from yeast using the two-hybrid system. The results showed that no interaction between the two proteins in yeast occurred (Figure 1). In addition, a XopJ C235A mutant in the conserved catalytic triad lost its ability to interact with RPT6 in yeast (Figure 1), indicating that the biochemical activity of XopJ might be required for its interaction with RPT6 in yeast.

Sequence identity between RPT6 from *N. tabacum* and the orthologous protein from the Xcv host plant pepper (*Capsicum annuum*) is 98% (Figure S1B) and a yeast two-hybrid analysis revealed binding of XopJ to the pepper RTP6 protein (Figure 1). Thus, RPT6 could represent a potential XopJ target protein during a compatible interaction of Xcv with pepper. However, given the high degree of sequence identity between the tobacco and pepper RTP6 we reasoned that further functional analysis of the XopJ/RPT6 interaction could be carried out with the tobacco NtRPT6.

### Interaction of XopJ and RPT6 *in planta* and *in vitro*

**Figure 2:**
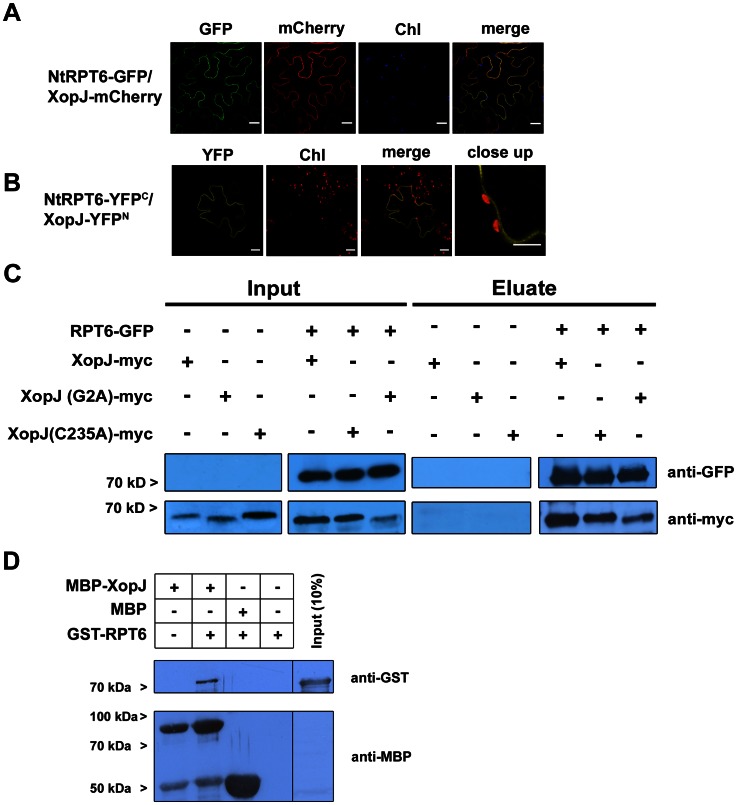
Subcellular localization of NtRPT6 and XopJ – NtRPT6 interaction *in planta*. (A) Subcellular localization of NtRPT6-GFP and XopJ-mCherry fusions in *N. benthamiana* leaves transiently transformed by Agro-infiltration. The green fluorescence (GFP), red fluorescence (mCherry) and chlorophyll autofluorescence (Chl) were monitored separately to prevent cross-talk of the fluorescence channels and the resulting fluorescence images were merged. Bars = 20 µm. Pictures show a representative result of at least three repetitions. (B) Visualization of protein interactions *in planta* by the BiFC assay. YFP confocal microscopy images show tobacco leaf epidermal cells transiently expressing constructs encoding the fusion proteins indicated. Merge indicates an overlay of the YFP and chlorophyll autofluorescence images. A close up of the same cells shows that the YFP fluorescence aligns with the PM. Bars = 20 µm. The experiment has been repeated three times with similar results. (C) Co-immunoprecipitation of NtRPT6-GFP with XopJ-myc and XopJ(C235A)-myc. NtRP6-GFP was transiently co-expressed in leaves of *N. benthamiana* using Agro-infiltration with either XopJ-myc or XopJ(C235A)-myc. After 48 h, total proteins (Input) were subjected to immunoprecipitation (Eluate) with GFP-Trap beads followed by immunoblot analysis using either anti-GFP or anti-myc antibodies. At least three repetitions with similar result have been conducted. (D) *In vitro* pull-down assay showing physical interaction of XopJ with RPT6. MBP-XopJ and GST-Rpt6 were expressed in *E. coli*. Pull down was performed using amylose resin. Proteins were detected in an immunoblot using antibodies as indicated.

Next, the subcellular localization of NtRPT6 was examined to determine whether it overlaps with that of XopJ in plant cells. Colocalization of both proteins would indicate that these proteins could interact *in planta*. The green fluorescent protein (GFP) was fused to the C-terminus of NtRPT6 and the fusion protein was expressed together with a XopJ-mCherry fusion protein, in leaves of *N. benthamiana* using Agrobacterium-infiltration. The fluorescence pattern was investigated using confocal laser scanning microscopy 48 h after infiltration. NtRPT6-GFP fluorescence was present at the plasma membrane (PM) and in the cortical cytoplasm (Figure 2A). XopJ was previously reported to localize to the PM involving N-myristoylation [19], [20]. Colocalization of NtRPT6-GFP with XopJ-mCherry revealed partially overlapping fluorescence patterns at the PM, indicating that both proteins could interact *in vivo* (Figure 2A).

To confirm the interaction of XopJ and NtRPT6 *in planta*, bimolecular fluorescence complementation (BiFC) assays were performed in *N. benthamiana* using transient expression via particle bombardment. XopJ and NtRPT6 were fused with the non-fluorescent N-terminal part of the yellow fluorescent protein (YFP^N^) and the C-terminal part of YFP (YFP^C^) at their C-termini, respectively. Homodimerization of cytosolic fructose-1,6-bisphosphatase in the cytosol served as a positive control (Figure S2). A combination of FBPase-YFP^N^ with NtRPT6-YFP^C^ or FBPase-YFP^c^ with NtRPT6-YFP^N^ induced no fluorescence (Figure S2). By contrast, strong YFP fluorescence was observed when a combination of XopJ-YFP^N^ with NtRPT6-YFP^C^ was expressed, demonstrating that XopJ/NtRPT6 interact in plant cells (Figure 2B). In accordance with a PM localization of XopJ, the YFP signal in XopJ/NtRPT6 BiFC experiments appeared to be confined to the PM as no fluorescence surrounding the chloroplasts could be detected which would be indicative for a cytosolic localization of the XopJ/NtRPT6 interaction (Figure 2B).

To further substantiate this finding a GFP-pull down assay was performed. To this end NtRPT6-GFP was transiently co-expressed with XopJ-myc in leaves of *N. benthamiana* using Agro-infiltration. Forty-eight hours after infiltration GFP-tagged NtRPT6 was pulled down from total leaf extracts using GFP-trap beads and the precipitate was subjected to western blot analysis with anti-GFP and anti-myc antibodies, respectively. As shown in Figure 2C NtRPT6-GFP was able to co-precipitate XopJ-myc which is indicative for an interaction between the two proteins *in planta*.

Previous results demonstrated that XopJ requires an intact catalytic triad and a functional myristoylation motif, respectively, to inhibit basal defence responses [19]. To further investigate structural requirements for the XopJ/NtRPT6 interaction *in planta*, a co-immunoprecipitation experiment was performed using the XopJ(C235A) mutant variety as well as the XopJ(G2A) variant. The results revealed that both mutant proteins retained their ability to interact with NtRPT6 in a pull-down experiment (Figure 2C) suggesting that biological activity and myristoylation, respectively, are *per se* not required for XopJ to interact with RPT6 *in planta*.

To exclude that the interaction between XopJ and RPT6 is mediated by a third eukaryotic protein an *in vitro* pull-down assay was performed. To this end, recombinant glutathione *S*-transferase (GST) tagged NtRPT6 was incubated with maltose-binding protein (MBP) tagged XopJ. A subsequent western blot revealed that GST-NtRPT6 was pulled down together with MBP-XopJ, demonstrating a direct physical interaction of both proteins which does not require additional factors (Figure 2D). MBP alone was not able to pull down GST-RPT6 and GST-RPT6 was not able to bind to the amylose matrix indicating specificity of the *in vitro* interaction (Figure 2D).

### XopJ suppresses proteasome activity

**Figure 3:**
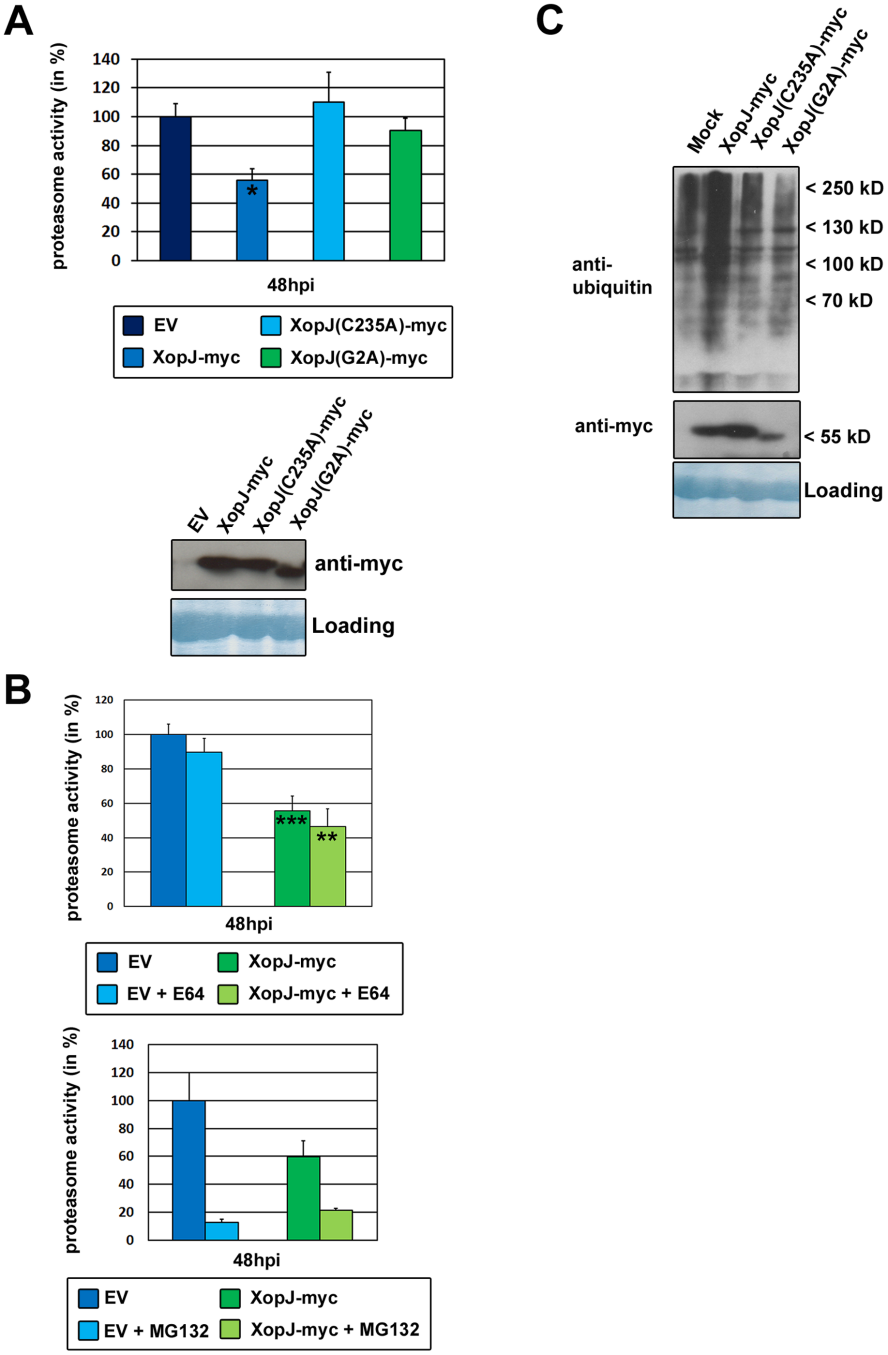
Transient expression of XopJ in *N.* *benthamiana* leaves inhibits proteasome activity. (A) Upper panel: Proteasome activity in *N. benthamiana* leaves transiently expressing XopJ-myc proteins. XopJ protein variants along with an empty vector (EV) control were transiently expressed in leaves of *N. benthamiana* using Agro-infiltration. After 48 h, relative proteasome activity in total protein extracts was determined by monitoring the breakdown of the fluorogenic peptide Suc-LLVY-AMC at 30°C in a fluorescence spectrophotometer. The empty vector (EV) control was set to 100%. Data represent the mean SD (n = 3). The experiment has been repeated more than three times with similar results. Lower panel: immunodetection of transiently expressed XopJ variants in the same leaves that were used for proteasome activity measurements. After immunodetection of proteins the membrane was stained with amido black to control for equal protein loading. (B) Upper panel: Proteasome activity in the presence of cysteine protease inhibitor E64 in *N. benthamiana* leaves transiently expressing XopJ-myc. XopJ protein along with an empty vector (EV) control was transiently expressed in leaves of *N. benthamiana* using Agro-infiltration. After 48 h, relative proteasome activity was determined by monitoring the breakdown of the fluorogenic peptide Suc-LLVY-NH-AMC at 30°C in a fluorescence spectrophotometer. Plant extracts were incubated with water or 100 µM E64 for 15 min at 30°C before measurements. The empty vector (EV) control was set to 100%. Data represent the mean SD (n = 4). Significant differences were calculated using Student's t-test and are indicated by: **, P<0.01; *** P<0.001. Lower panel: Proteasome inhibition by MG132. XopJ and empty vector (EV) control were transiently expressed in leaves of *N. benthamiana* using Agro-infiltration. After 48 h, relative proteasome activity was determined by monitoring the breakdown of the fluorogenic peptide Suc-LLVY-NH-AMC at 30°C in a fluorescence spectrophotometer. Plant extracts were incubated with 1% EtOH or 50 µM MG132 for 15 min at 30°C before measurements. The empty vector (EV) control was set to 100%. Data represent the mean SD (n = 3). (C) Distribution of ubiquitin conjugates in *N. benthamiana* leaves transiently expressing XopJ-myc proteins. Total proteins were extracted 48 h after infiltration with Agrobacteria harbouring the respective XopJ expression constructs. Ubiquinated proteins were detected using an anti-ubiquitin antibody. Expression of the XopJ variants was verified using an anti myc-antibody. After immunodetection of proteins the membrane was stained with amido black to control for equal protein loading.

After having established that XopJ and RPT6 interact in plant cells we next sought to investigate whether this interaction exerts any effect on overall proteasome activity in plants. To this end, the proteasome activity was monitored using a fluorogenic peptide (Suc-LLVY-AMC) which is a substrate for the chymotrypsin-like activity of the proteasome. This substrate has been shown to be split by the 26S proteolytic complex, whereas the 20S proteasome, which is not involved in the degradation of ubiquitinated proteins, is known to have no activity for the peptide breakdown [23]. As shown in Figure 3A, transient expression of XopJ-myc in leaves of *N. benthamiana* led to a reduction in proteasome activity of approximately 40% as compared to leaves infiltrated with the empty vector control. Mutation of the catalytic triad in XopJ(C235A) as well as a mutation in the myristoylation site in the XopJ(G2A) variant abolished the inhibitory effect on proteasome activity although both proteins were expressed to comparable levels as wild-type XopJ (Fig 3A). This indicates that XopJ requires an intact catalytic triad and proper localization to the PM to affect proteasome activity. Furthermore, an unrelated T3E from Xcv, XopB, did not inhibit proteasome activity upon transient expression (Figure S3), indicating that inhibition of the proteasome is not a general feature of type III effectors.

Previous reports suggest that Suc-LLVY-AMC could also be cleaved by cysteine proteases in addition to serving as a substrate for the proteasome [24]. To rule out that XopJ inhibits cysteine proteases, the proteasome activity assay was performed in the presence or absence of the broad spectrum cysteine protease inhibitor E64. As shown in Figure 3B, E64 caused a reduction of Suc-LLVY-AMC cleaving activity of approximately 10% in extracts prepared from control leaves as well as in extracts prepared from *N. benthamiana* leaves transiently expressing XopJ. Thus, the inhibitory effect of XopJ on the proteasome activity is not affected by the addition of E64. In turn, when extracts from control leaves were assayed in the presence of the potent proteasome inhibitor MG132 Suc-LLVY-AMC cleaving activity was reduced by approximately 85% indicating a high degree of specificity of the assay (Figure 3B). XopJ was not able to inhibit proteasome activity any further than what was observed in the presence of MG132 (Figure 3B). These data strongly suggest that XopJ specifically inhibits the chymotrypsin-like activity of the proteasome and not cysteine proteases in general.

To determine whether the reduced proteasome activity would lead to the accumulation of ubiquinated proteins a western blot using an anti-ubiquitin antibody was performed on total protein extracts from leaves transiently expressing XopJ-myc or the XopJ(C235A)-myc and XopJ(G2A)-myc variants, respectively. As shown in Figure 3C, a high-molecular weight smear diagnostic for the accumulation of non-degraded poly-ubiquinated proteins was visible in extracts from XopJ-myc expressing leaves. Although there is some variation in staining intensity, expression of the mutated XopJ variants did not cause a comparable accumulation of ubiquitin-decorated proteins (Figure 3C). This is in line with the observation that these proteins do not affect proteasome activity upon transient expression.

### XopJ contributes to bacterial multiplication at late stages of infection and attenuates proteasome activity during infection of pepper by virulent Xcv

**Figure 4:**
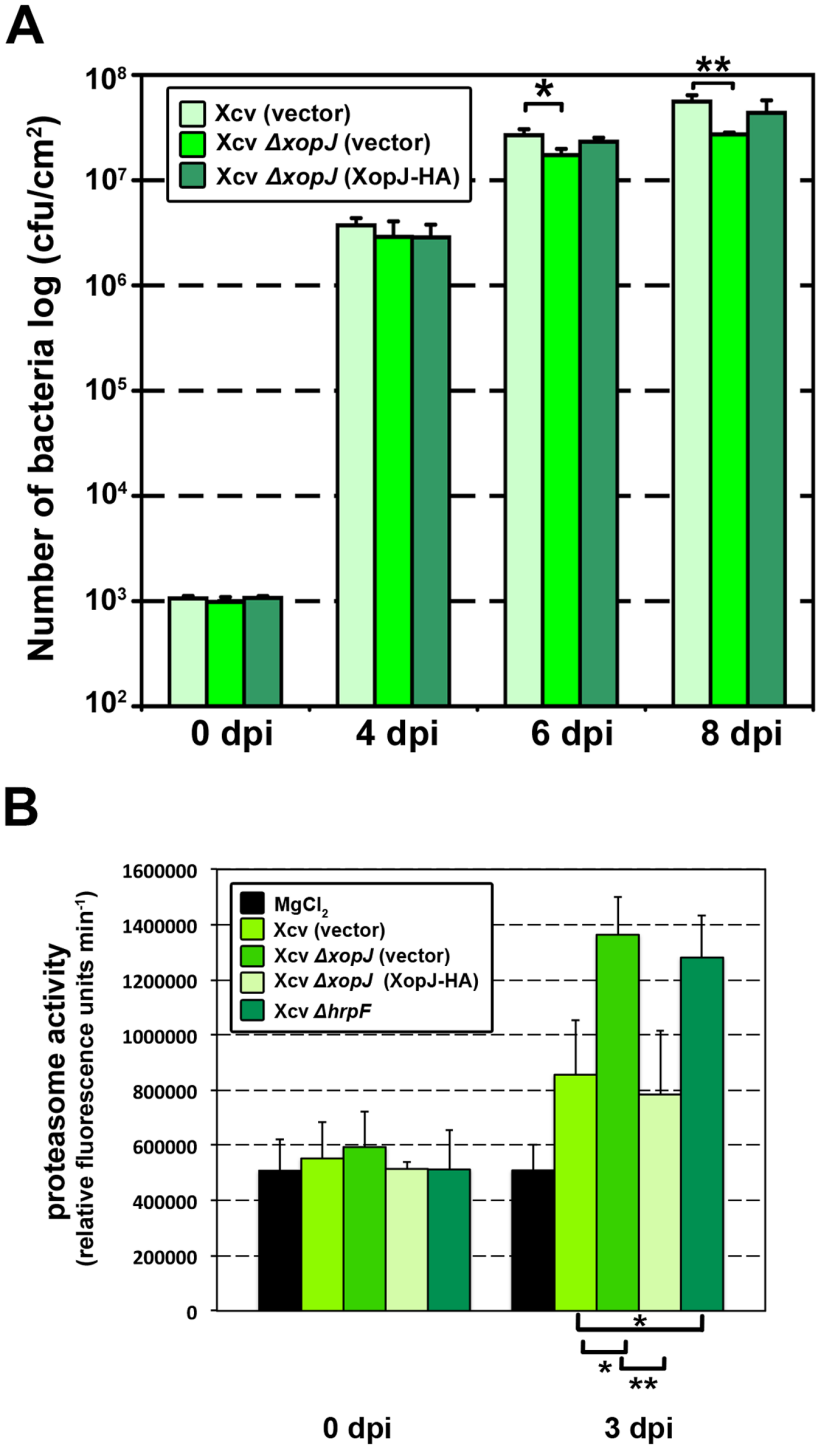
XopJ contributes to bacterial growth in pepper at late stages of infection and dampens proteasome activity in infected pepper leaves. (A) Growth of Xcv 85-10 (vector), Xcv *ΔxopJ*, Xcv *ΔxopJ (XopJ)*, strains in pepper ECW leaves. Leaves were hand-infiltrated with a 10^5^ cells/ml suspension of bacteria. The number of bacteria in each leaf was quantified at 0, 4, 6, 8 dpi. Data represent the mean SD (n = 3). Significant differences were calculated using Student's t-test and are indicated by: *, P<0.05; **, P<0.01. The experiment was repeated three times with almost identical results. A representative result is shown. (B) XopJ reduces proteasome activity during Xcv-pepper interaction. Leaves were infiltrated with strains indicated in the figure. After 3 dpi proteasome activity in total leaf extracts was determined by monitoring the breakdown of the fluorogenic peptide suc-LLVY-NH-AMC at 30°C in a fluorescence spectrophotometer. Data represent the mean SD (n = 4). Significant differences were calculated using Student's t-test and are indicated by: *, P<0.05; **, P<0.01. A representative result of more than three repetitions with independent sets of plants is shown.

The results obtained thus far indicate that XopJ and RPT6 interact in yeast and when transiently expressed in leaves of *N. benthamiana*. This interaction somehow leads to a reduction in proteasome activity interfering with the turnover of ubiquinated proteins. Thus, the question arises as to whether XopJ has a similar effect when translocated in a type III dependent manner during a compatible interaction and how this could contribute to bacterial virulence. It has previously been shown that an Xcv mutant strain of XopJ was not affected in bacterial growth and symptom formation on susceptible plants, indicating subtle contributions to bacterial virulence or functional redundancy [17]. The mutant strain analyzed in that study contained a frameshift mutation in *xop*J. In the present study, we constructed a *xop*J null mutant (designated Xcv Δ*xop*J) in Xcv strain 85–10 by deleting 880 nt within the XopJ coding region by homologous recombination. To re-assess the contribution of XopJ to bacterial multiplication, susceptible pepper plants were infiltrated with the Xcv Δ*xop*J strain and with the Xcv wild type control (each carrying the broad host range vector pBBR1 MCS-5 [25] that was subsequently used for complementation) at a bacterial titer of 10^5^ cfu/ml. Xcv Δ*xop*J multiplication was slightly but significantly reduced as compared to the control at 6 dpi (days post infection) and 8 dpi (Figure 4A). Xcv Δ*xop*J strains carrying the broad host vector pBBR1 MCS-5 containing the *xop*J ORF tagged with HA to facilitate immunological detection [Xcv Δ*xop*J (xopJ-HA)] exhibited wild-type growth at 6 dpi and 8 dpi (Figure 4A). This indicates that XopJ is required for maximal Xcv growth in susceptible pepper leaves at the late stages of infection.

Measurement of the overall leaf proteasome activity three days after infection provides evidence that Xcv wild-type causes a significant induction of proteasome activity (Figure 4B). The rise in activity was significantly higher with Xcv Δ*xop*J (vector) and also significantly induced compared to leaves infected with Xcv Δ*xop*J (XopJ-HA) or Xcv (vector), indicating that XopJ is necessary to dampen the proteasome activity *in vivo* (Fig. 4B). Pepper plants infected with Xcv Δ*hrpF*, a T3SS deficient mutant that is not able suppress basal defence responses, displayed elevated proteasome activity 3 dpi, suggesting that the proteasome activity is induced during basal defence (Fig. 4B). These data demonstrate that XopJ is able to reduce proteasome activity after translocation by a virulent Xcv in a type III dependent manner.

### XopJ leads to suppression of cell death during a compatible interaction

**Figure 5:**
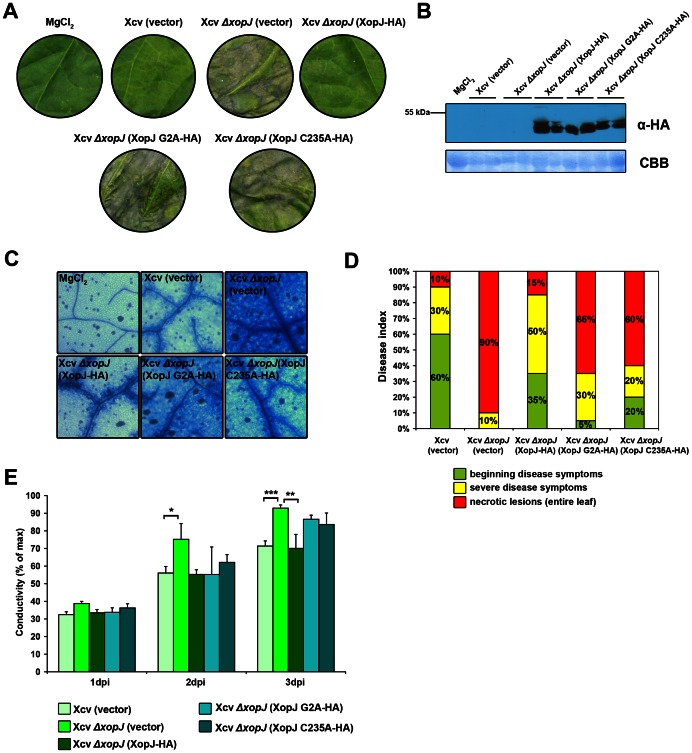
XopJ suppresses cell death during the compatible Xcv-pepper interaction. (A) Xcv (vector), Xcv *ΔxopJ* (vector), Xcv *ΔxopJ* (XopJ-HA), XopJ (G2A-HA) and (C235A-HA) were inoculated at a bacterial density of 2×10^8^ cfu ml^−1^ into leaves of pepper ECW plants. Leaf phenotype was photographed at 3 d post infection (dpi). (B) Protein extracts from pepper leaves infiltrated with 1 mM MgCl_2_, Xcv (vector), Xcv *ΔxopJ* (vector), Xcv *ΔxopJ* (XopJ-HA), XopJ (G2A-HA) and (C235A-HA) at 3 dpi were prepared. Equal volumes representing approximately equal protein amounts of each extract were immunoblotted and proteins were detected using anti-HA antiserum. (C) Trypan blue staining of infected tissue of ECW plants reveals reduced cell death in the presence of functional XopJ. Xcv (vector), Xcv *ΔxopJ* (vector), Xcv *ΔxopJ* (XopJ-HA), XopJ (G2A-HA) and (C235A-HA) were inoculated at a bacterial density of 2×10^8^ cfu ml^−1^ into leaves of pepper ECW plants. Samples of infected and untreated leaves were taken 3 dpi and stained with trypan blue. Dead plant cells stain blue. Grey and black spots represent calcium oxalate crystals. (D) Summary of observed phenotypes. 20 pepper plants were infected with the different Xcv strains indicated in this figure and then scored for phenotype development at 3 dpi. (E) Delivery of XopJ by Xcv leads to reduced ion leakage in pepper. Ion leakage was measured in pepper plants infected with. Xcv (vector), Xcv *ΔxopJ* (vector), Xcv *ΔxopJ* (XopJ-HA), XopJ (G2A-HA) and (C235A-HA). Conductivity was measured at the time points indicated. Data represent the mean SD (n = 3). Significant differences are indicated by asterisks (* P<0.05; ** P<0.01; *** P<0.001) and were calculated using Student's t-test.

Given the fact that the *in planta* growth as a measure for virulence was affected in a Xcv Δ*xop*J mutant strain at late stages of infection we searched for XopJ phenotypes that could provide a link between effector function and virulence activity. To this end we assessed phenotype development and symptom production after infection of susceptible pepper plants with a high bacterial titre (10^8^ cfu/ml) of the Xcv Δ*xop*J mutant as compared to the wild type strain. Leaves inoculated with the Xcv Δ*xop*J deletion strain developed necrotic lesions 3 dpi while those infected with the Xcv (vector) strain remained symptomless at the same time point (Figure 5A). However, 5 dpi a similar degree of necrotic lesions observed for Xcv (vector) infected leaves as in the Xcv Δ*xop*J mutant at 3 dpi (Figure S5). This suggests that a deletion of XopJ affects the kinetics of symptom development during infection by inhibiting the onset of necrosis. Introduction of XopJ (G2A-HA) and (C235A-HA) constructs into the Xcv *Δxop*J null mutant did not abolish necrosis induction, suggesting that a functional XopJ is necessary and sufficient to suppress development of necrosis in pepper (Figure 5A). Proper expression of all XopJ variants was verified by western blotting using the HA-tag (Figure 5B). The timing of tissue necrosis is slow compared with the rapid, localized hypersensitive cell death response characteristic of R protein–mediated defences in resistant hosts. Therefore, the necrotic phenotype observed in leaves infected with the Xcv Δ*xop*J strain rather reflects normal but accelerated symptom development associated with later stages of disease [26].

To strengthen the idea that XopJ suppresses the onset of necrosis, we performed an *in planta* mixed-inoculum experiment by first infiltrating Xcv *Δxop*J harbouring the XopJ-HA construct into pepper and, with a time shift of three hours, Xcv Δ*xop*J. As shown in Figure S6, cell death was not induced in this mixed-inoculum experiment, indicating that XopJ suppresses necrosis in the Xcv-pepper interaction. To characterize the suppression of cell death by XopJ in more detail, plant cell death was monitored by trypan blue staining of the infected tissue. Trypan blue is a vital stain that specifically stains dead cells but is not absorbed by cells with intact plasma membranes [27]. Xcv (vector), *Δxop*J (vector) and the XopJ-complemented strains were inoculated into leaves of pepper plants. Samples from infected leaf tissue were collected 3 dpi, stained with trypan blue and analyzed by transmission light microscopy. In contrast to untreated leaf material that remained unstained, almost all cells were stained by trypan blue in tissue infected with Xcv Δ*xop*J null mutant (Figure 5C). In leaves inoculated with Xcv harbouring the empty vector or Xcv *Δxop*J (XopJ-HA), only a few cells were stained, whereas Xcv *Δxop*J XopJ (G2A-HA) and (C235A-HA) strains displayed enhanced staining (Figure 5C). In summary, this result is in accordance with the macroscopically observed phenotype caused by the different Xcv strains on pepper leaves, summarized in Figure 5D. The disease index demonstrates that the phenotypes caused by the different Xcv strains consistently occur in a population of 20 individual pepper ECW plants, e.g., Xcv strains lacking XopJ induced tissue necrosis in 90% of infected pepper plants (Figure 5D).

In order to quantify cell death elicitation by the different Xcv strains, we determined ion leakage induced by all strains. Cell death is often preceded by an enhanced ion leakage in dying cells due to membrane damage and thus provides a quantitative measure of cell death-associated phenotypes. As expected for an ongoing cell death, conductivity significantly increased at 3 dpi in samples infiltrated with Xcv *Δxop*J, Xcv *Δxop*J XopJ (G2A-HA) and (C235A-HA) in comparison to Xcv (vector) and Xcv *Δxop*J (XopJ-HA), which is in agreement with the observed phenotypes (Figure 5E). Thus, in susceptible pepper leaves, XopJ action does promote Xcv multiplication by slowing down the rate of secondary symptom development such as tissue necrosis.

### Reduced proteasome activity delays cell death induction

**Figure 6:**
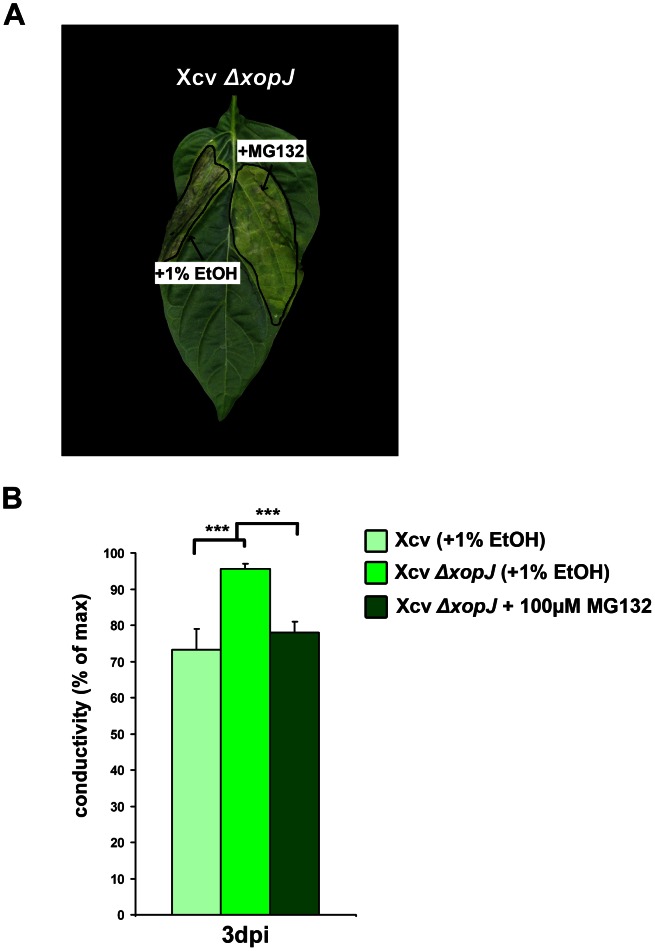
The proteasome inhibitor MG132 prevents development of necrosis in Xcv Δ*xopJ* infected pepper leaves. (A) Xcv Δ*xopJ* with 100 µM MG132 or 1% EtOH (control) were inoculated at a bacterial density of 2×10^8^ cfu ml^−1^ into leaves of pepper ECW plants. Plant reactions were photographed at 3 d post infection (dpi). (B) MG132 treatment leads to a reduced ion leakage in pepper in the absence of XopJ. Ion leakage was measured in pepper plants infected with Xcv, Xcv Δ*xopJ* with MG132 or 1% EtOH. Conductivity was measured at the time point indicated. Data represent the mean SD (n = 3). Significant differences are indicated by asterisks (*** P<0.001) and were calculated using Student's t-test.

Given that XopJ prevents necrosis and depletes proteasome activity during a compatible interaction of Xcv with pepper, we sought to investigate whether both events during infection are connected with each other. To this end a pharmacological approach to determine whether the inhibition of the proteasome activity could account for XopJ-mediated suppression of cell death was taken. When Xcv Δ*xop*J was co-infiltrated with the well-characterized proteasome inhibitor, MG132 (100 µM in 1% ethanol), less necrotic lesions compared to control treatment could be observed, indicative for a complementation of the loss of XopJ by MG132 (Figure 6A). Ion leakage measurements were also consistent with the observed phenotype, as leaves co-infiltrated with Xcv Δ*xop*J and MG132 exhibited significantly reduced conductivity compared to the control treatment and being similar to Xcv WT induced ion leakage (Figure 6B). Thus, we conclude that MG132 can phenocopy XopJ function leading to the suppression of cell death. Taken together, these findings suggest a connection between inhibition of the proteasome by XopJ and its ability to prevent cell death induction during disease development.

### XopJ reduces SA pools during infection

**Figure 7:**
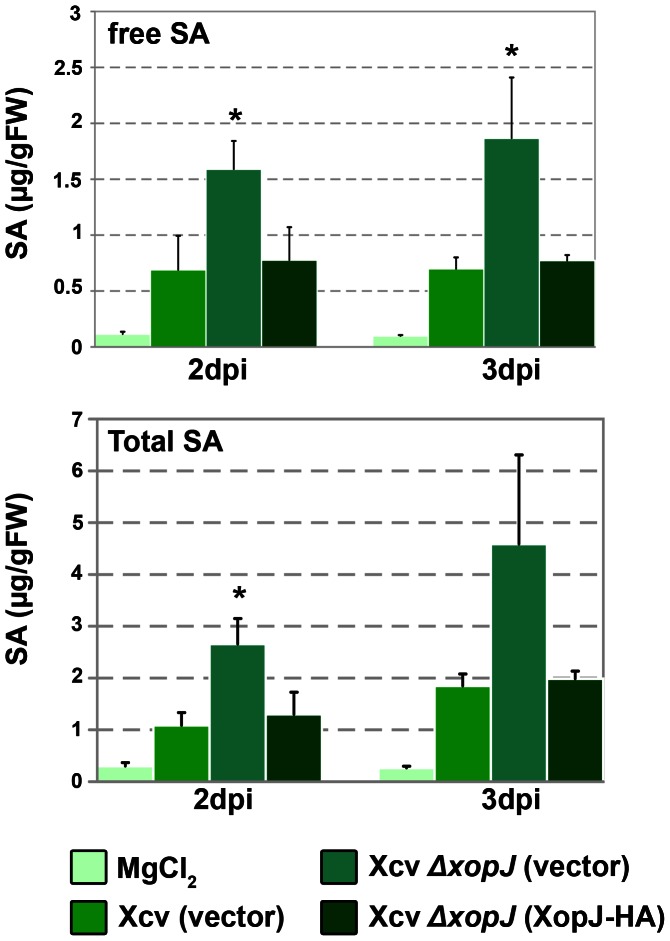
XopJ reduces levels of free and conjugated SA in leaves of Xcv infected pepper plants. Susceptible pepper ECW leaves were hand-inoculated with MgCl_2_ or a 2×10^8^ cfu/mL suspension of Xcv (vector), Xcv Δ*xopJ* (vector) and Xcv Δ*xopJ* (XopJ). Free SA and total SA (free SA+SAG) levels in infected tissue were measured 2 and 3 dpi. Data represent the mean SD (n = 3). Significant differences are indicated by asterisks (* P<0.05) and were calculated using Student's t-test. FW, Fresh weight.

It has previously been shown that tissue necrosis associated with the secondary phase of Xcv infection of tomato leaves is dependent on the phytohormone salicylic acid (SA) [26]. Furthermore, experiments by Kim et al. [28] suggest that the Xcv effector XopD is able to suppress SA responses and plant immunity providing a paradigm for a Xcv T3E that interferes with hormonal defence. This finding prompted us to investigate whether XopJ could influence SA levels in infected pepper plants. To this end, SA pools (free and conjugated SA) were quantified in susceptible pepper plants 2 and 3 dpi infected with a high titre of Xcv (10^8^ cfu/ml). By two and 3 dpi, leaves inoculated with Xcv *Δxop*J had approximately two-fold more free and total SA than leaves infected with Xcv wildtype (Figure 7). Complementation of Xcv *Δxop*J with XopJ resulted in SA levels comparable to Xcv WT infected leaves. The decrease in the pool of SA hence indicates that XopJ significantly diminishes the magnitude of SA accumulation in Xcv infected pepper plants (Figure 7).

If the function of XopJ is to inhibit symptom development through interference with SA accumulation, then application of SA to wild type Xcv infected pepper leaves may mimic the phenotype of an Xcv Δ*xop*J deletion mutant. Indeed, when Xcv wild type infected pepper leaves were sprayed with 5 mM SA 2 dpi necrotic lesions developed 3 dpi that were comparable to those observed on Xcv Δ*xop*J infected leaves at the same time point without SA treatment (Figure S7). Thus, Xcv infected tissue remains sensitive to exogenously applied SA even in the presence of XopJ.

### XopJ alters SA- and senescence-dependent gene expression during infection

**Figure 8:**
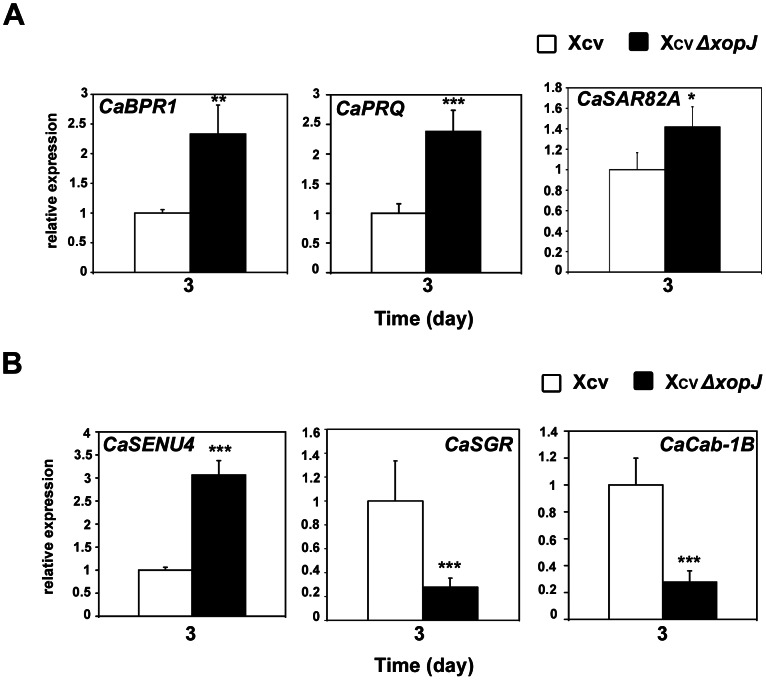
XopJ alters the mRNA abundance of senescence- and SA-dependent genes. Total RNA was isolated from pepper leaves infiltrated with 2×10^8^ cfu/mL of Xcv or Xcv *ΔxopJ*, respectively. Quantitative real-time RT-PCR was performed to monitor mRNA levels. (A) SA-dependent-upregulated genes. (B) senescence-downregulated genes (*CaSGR* and *CaCab-1B*) and senescence-upregulated genes (*CaSENU4*). In both panels relative expression levels at 3 dpi are shown. *Actin* expression was used to normalize the expression value in each sample, and relative expression values were determined against the average value of the sample infected with wild- type Xcv. Leaf material from 4 independent pepper plants was pooled and analyzed in triplicates. Data represent the mean SD. Significant differences were calculated using Student's t-test and are indicated by: *, P<0.05; **, P<0.01; ***, P<0.001.

After having established that XopJ negatively affects SA levels during infection, we next used quantitative real-time RT-PCR (qPCR) to investigate whether SA-dependent gene expression is impaired in plants infected with Xcv lacking XopJ. Thus, we monitored the impact of Xcv *Δxop*J infection in comparison to Xcv WT infection on the mRNA levels of the SA marker genes *CaBPR1* (basic PR1 protein) *CaPR-Q* (chitinase) and *CaSAR82A* (SAR8.2) ([29]–[31]. The mRNA levels of all three SA-inducible marker genes were significantly elevated in pepper leaves infected with Xcv Δ*xop*J 3 dpi when compared to leaves infected with Xcv wild type bacteria (Figure 8A). This is consistent with the notion that XopJ leads to reduced SA pools during infection that then leads to an altered SA-dependent gene expression in pepper.

Pathogen infection has previously been shown accelerate the onset of leaf senescence and evidence suggests that this is at least in part mediated by SA signalling [28], [32], [33]. Based on the phenotype of pepper leaves infected with the Xcv Δ*xop*J strain we hypothesized that XopJ could delay senescence-associated processes during infection. Thus, qPCR was used to analyze mRNA levels of genes whose expression levels significantly change during age- and pathogen- induced senescence. Three genes were analyzed: *CaSENU4* encodes a pathogenesis-related protein 1b1 that is induced in response to aging and SA [34], [35]. *CaSGR* (STAYGREEN) and *CaCab-1b* (chlorophyll binding protein) are senescence markers because their expression has been shown to decrease during senescence in tomato leaves [28], [35], [36]. As shown in Figure 8B transcripts of *CaSGR* and *CaCab-1b* were significantly down regulated in tissue infected with Xcv *Δxop*J, compared to Xcv WT infection, while mRNA levels of *CaSENU4* were significantly up-regulated, indicating that pepper leaves display accelerated senescence when infected with a Xcv Δ*xop*J strain as compared to Xcv wild type infected leaves. Thus, XopJ seems to suppress senescence-associated gene expression likely to delay symptom development and tissue necrosis during later stages of infection.

### SA induces proteasome activity

**Figure 9:**
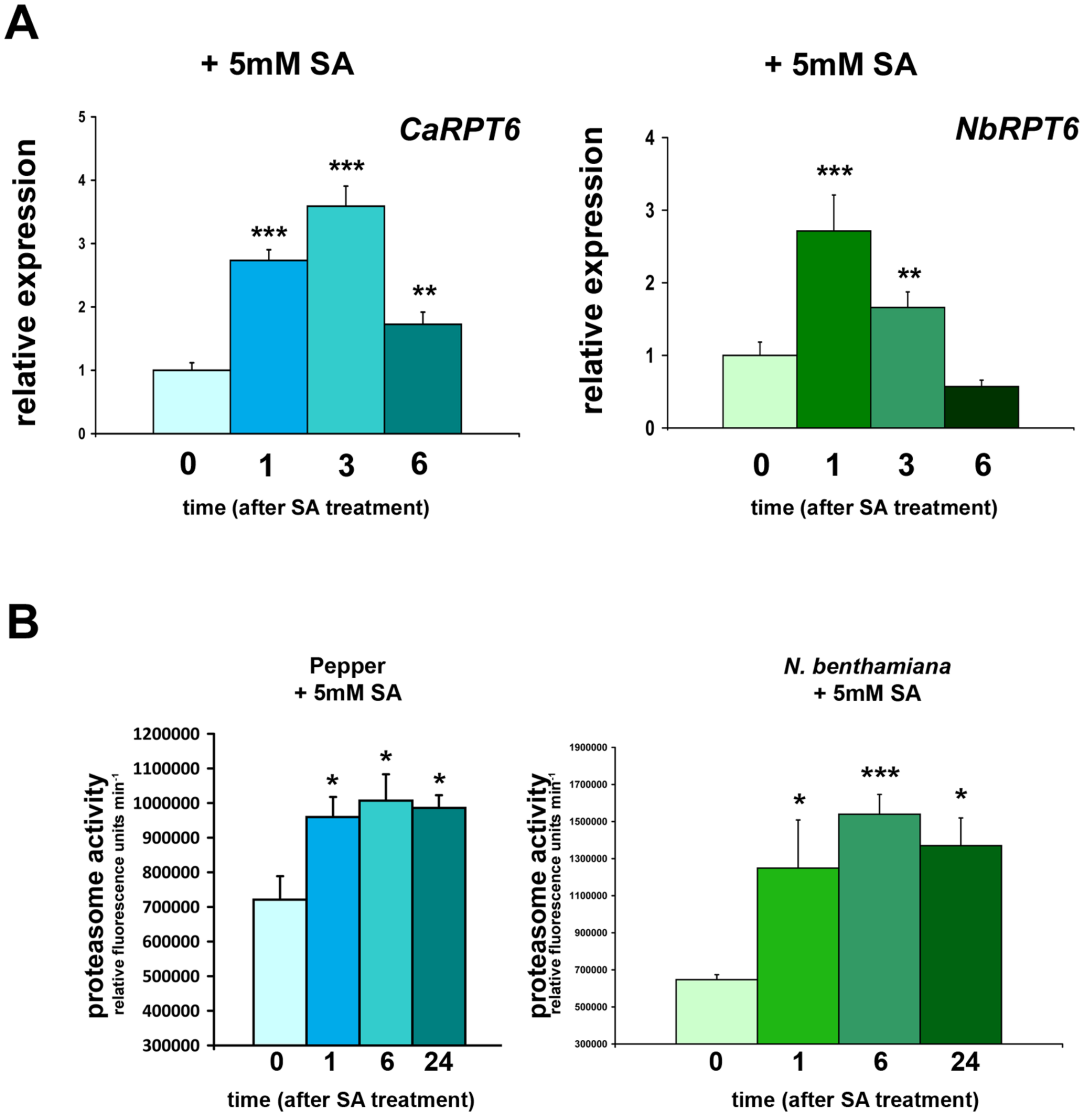
SA treatment induces proteasome activity and RPT6 gene expression in *N.* *benthamiana* and pepper ECW plants. (A) Pepper and *N. benthamiana* leaves were treated with 5 mM SA. Total RNA was isolated at 0 (untreated), 1, 3, 6 hour after SA application. Q-PCR was performed to monitor *CaRPT6* and *NbRPT6* mRNA levels. Relative expression levels at time points indicated are shown. *Actin* expression was used to normalize the expression value in each sample, and relative expression values were determined against the average value of the untreated sample. Data represent the mean SD (n = 4). Significant differences were calculated using Student's t-test and are indicated by: *, P<0.05; **, P<0.01; ***, P<0.001. (B) Pepper and *N. benthamiana* leaves were sprayed with 5 mM SA and proteasome activity in total leaf extracts was determined at time points indicated in the figure by monitoring the breakdown of the fluorogenic peptide sLLVY-NH-Mec at 30°C in a fluorescence spectrophotometer. Data represent the mean SD (n = 3) Significant differences are indicated by asterisks (* P<0.05; *** P<0.001) and were calculated using Student's t-test.

Since XopJ is required to suppress cell death correlating with reduced proteasome activity and decreased SA levels, we sought to investigate whether SA would have an influence on proteasome activity and gene expression of the proteasome subunit RPT6. To test this, pepper and *N. benthamiana* leaves were sprayed with 5 mM SA and the transcript abundance of *RPT6* in response to SA was determined. Transcript levels of *CaRPT6* and *NbRPT6* were significantly elevated about threefold 3 h after SA treatment (Figure 9A). We then determined whether the proteasome activity is also induced after SA treatment. Measurements revealed that proteasome activity is significantly induced by SA, up to 40% in pepper and up to 130% in *N. benthamiana*, having a peak at 6 h after SA application (Figure 9B). These data demonstrate that elevated proteasome activities and up-regulation of *RPT6* gene expression occurs in response to activation of the SA signalling pathway.

Since SA levels increase during a compatible interaction of Xcv with pepper, we next analysed *RPT6* gene expression during infection. To this end, we monitored *RPT6* mRNA levels in pepper leaves infiltrated with 1 mM MgCl_2_, Xcv and Xcv *Δxop*J (10^8^ cfu/ml). At 3 dpi Xcv WT significantly elevated *RPT6* gene expression in comparison to MgCl_2_ (Figure S8). Intriguingly, the increase in transcript abundance was even higher in Xcv *Δxop*J infected pepper tissue displaying a significant difference in comparison to Xcv-infected tissue. As RPT6 is an SA inducible gene (Figure 9A) and plants infected with Xcv *Δxop*J display significantly higher SA pools than Xcv WT infected pepper leaves (Figure 7), we conclude that *RPT6* gene expression during infection depends on the phytohormone SA.

### Virus – induced gene silencing of *NPR1* compromises XopJ-dependent phenotypes in pepper

**Figure 10:**
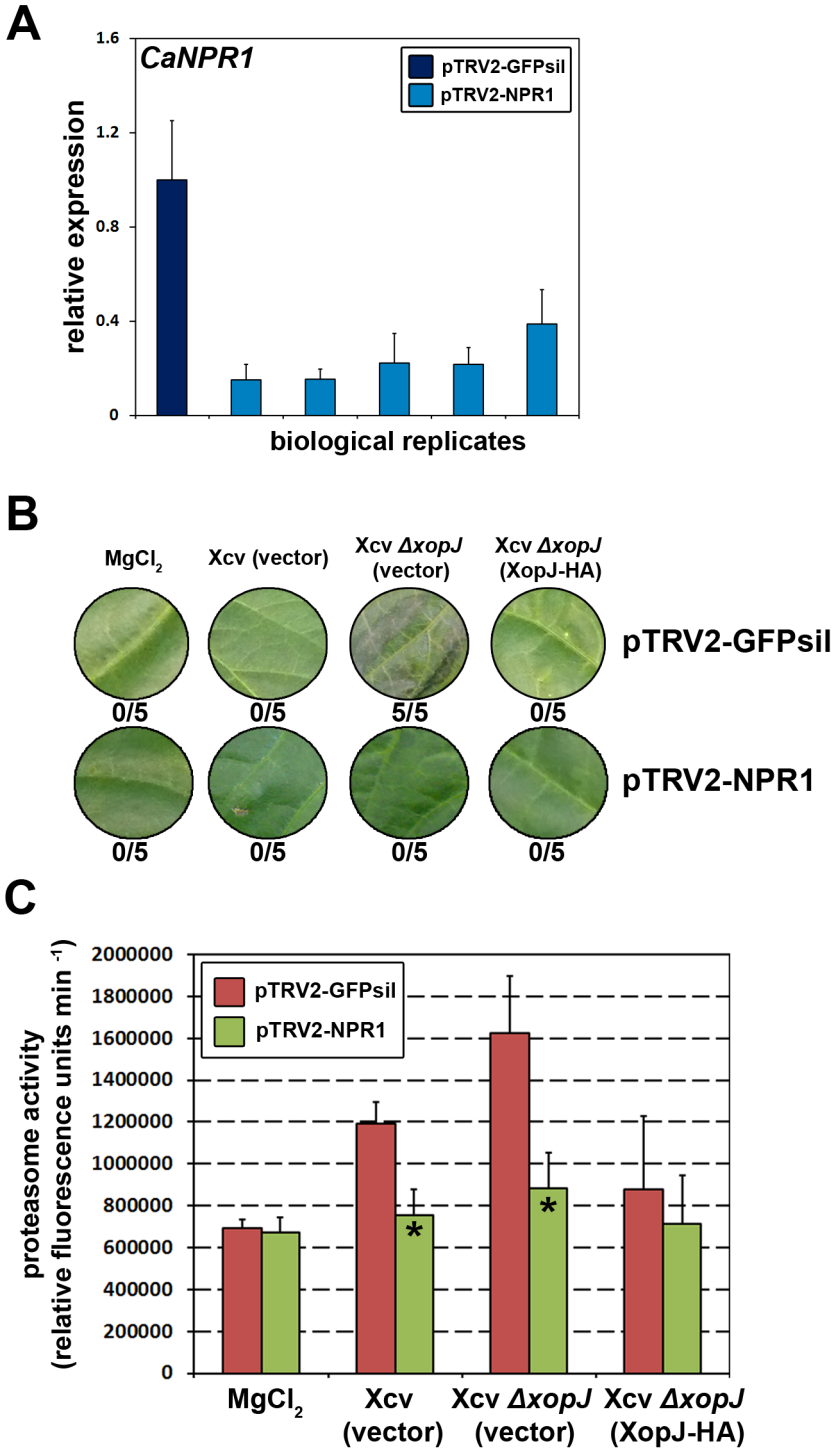
VIGS of *NPR1* interferes with XopJ-dependent phenotypes in pepper. (A) qRT-PCR analysis of NPR1 mRNA level in *NPR1* silenced pepper plants. Relative expression levels at 21 dpi are shown. *Actin* expression was used to normalize the expression value in each sample, and relative expression values were determined against pTRV2-GFPsil plants. (B) Xcv (vector), Xcv *ΔxopJ* (vector), Xcv *ΔxopJ* (XopJ-HA) were infiltrated at a bacterial density of 2×10^8^ cfu ml^−1^ into leaves of pTRV2-GFPsil and pTRV2-NPR1 plants. Plant reactions were photographed at 3 d post infection (dpi). The number of leaves showing necrosis is indicated below the appropriate construct. (C) Leaves were infiltrated with strains indicated in the figure. At 3 dpi proteasome activity in total leaf extracts was determined by monitoring the breakdown of the fluorogenic peptide suc-LLVY-NH-AMC at 30°C in a fluorescence spectrophotometer. Data represent the mean SD (n = 3). Significant differences were calculated using Student's t-test and are indicated by: *, P<0.05.

In an approach to provide more direct evidence for a connection between XopJ-mediated perturbations of the proteasome and SA-signalling, we used virus-induced gene silencing (VIGS) in pepper with *Tobacco rattle virus* (TRV), followed by infection with different Xcv strains. NPR1 is a key positive regulator of SA-mediated defence responses notably by activating transcription of a battery of genes in response to rising SA-levels [37]. To investigate the involvement of SA-signalling via NPR1 pepper seedlings were infiltrated with a mixture of *A. tumefaciens* strains of pTRV1 (CaMV 35S-driven TRV RNA1) and pTRV2-NPR1 (TRV RNA2 containing the target sequence), or pTRV2-GFPsil (serving as a control for infection symptoms). Three weeks after TRV inoculation, silencing of the target gene was confirmed by qRT-PCR (Figure 10A). Subsequently, plants were infiltrated with either Xcv, Xcv *Δxop*J or Xcv *Δxop*J (XopJ-HA). At 3 dpi silencing of *NPR1* gene expression prevented development of host cell necrosis in leaves infected with the Xcv strain lacking XopJ while pTRV2-GFPsil leaves were necrotic (Figure 10B). The fact that XopJ-dependent necrosis does not occur in plants defective in *NPR1* expression suggests a direct involvement of SA-signalling in XopJ-dependent phenotype development.

As previously observed measurement of the proteasome activity 3 dpi in pTRV2-GFPsil plants revealed increased activity in Xcv and Xcv Δ*xop*J infected leaves as compared to the mock-infiltrated control with a more pronounced rise in Xcv Δ*xop*J infected leaves confirming the inhibitory effect of XopJ on the proteasome during infection (Figure 10C). By contrast, in plants silenced for *NPR1* expression the proteasome activity was only slightly increased as compared to the mock-infected control with a significantly lower rise than in pTRV2-GFPsil plants (Figure 10C). This shows that plants impaired in SA signalling are unable to induce proteasome activity upon infection and opens the possibility that activation of the proteasome by SA could be mediated through NPR1.

### Proteasome activity is required for basal defence

Although an important role of the ubiquitin-proteasome system during the regulation of plant immune responses is increasingly recognized, a direct link between proteasome activity and basal defence is not firmly established [38]. Effectors inhibit basal defence at different levels such as PAMP-signalling, transcription and translation of defence related genes or vesicle trafficking and cell wall associated defence responses. Thus, we first attempted to narrow down the level on which XopJ could interfere with PTI. To determine the effect of XopJ on PAMP signalling we investigated MAPK activation followed by flg22 treatment in seedlings of transgenic Arabidopsis plants expressing XopJ under control of the ethanol-inducible promoter [19]. Ethanol-induced seedlings were treated with flg22 and MAPK activation was monitored using the phospho-p44/p42 antibody that specifically recognizes the active phosphorylated MAPK form. Western blot analysis revealed that ethanol-induced XopJ plants displayed no difference in MAPK activation in response to flg22 treatment as compared to non-induced alc-XopJ control plants (Figure S8A). Measurement of the proteasome activity in these plants showed a clear reduction in alc-XopJ plants after induction with ethanol (Figure S8B). Thus, XopJ flg22 induced MAPK signalling in Arabidopsis seems not to be affected by a XopJ mediated reduction in proteasome activity.

In a more direct attempt to study the involvement RPT6/proteasome function in basal defence we performed (VIGS) to reduce *RPT6* mRNA levels in pepper plants. However, two to three weeks after infection with the TRV silencing constructs tissues of RPT6 silenced plants began to collapse and the plants died shortly after (Figure S9). The lethal phenotype of RPT6-VIGS plant precludes further genetic analysis of RPT6 function during defence but highlights the essential function of this proteasome subunit for cell viability.

We thus decided to follow a pharmacological approach using the specific proteasome inhibitor MG132 to study the impact of reduced proteasome activity on basal defence responses. XopJ has previously been shown to inhibit secretion of a secGFP reporter. In addition, ethanol-inducible XopJ expression in leaves of transgenic Arabidopsis strongly compromises in the ability to deposit callose associated with papillae, a hallmark of cell wall–associated defence, in response to inoculation with the nonpathogenic *P. syringae* pv. tomato DC3000 *hrcC* mutant [19]. In order to investigate whether the same effects could be mediated by inhibition of the proteasome, *N. benthamiana* leaves transiently expressing secGFP were infiltrated with MG132 and analyzed for GFP fluorescence using confocal microscopy.

**Figure 11:**
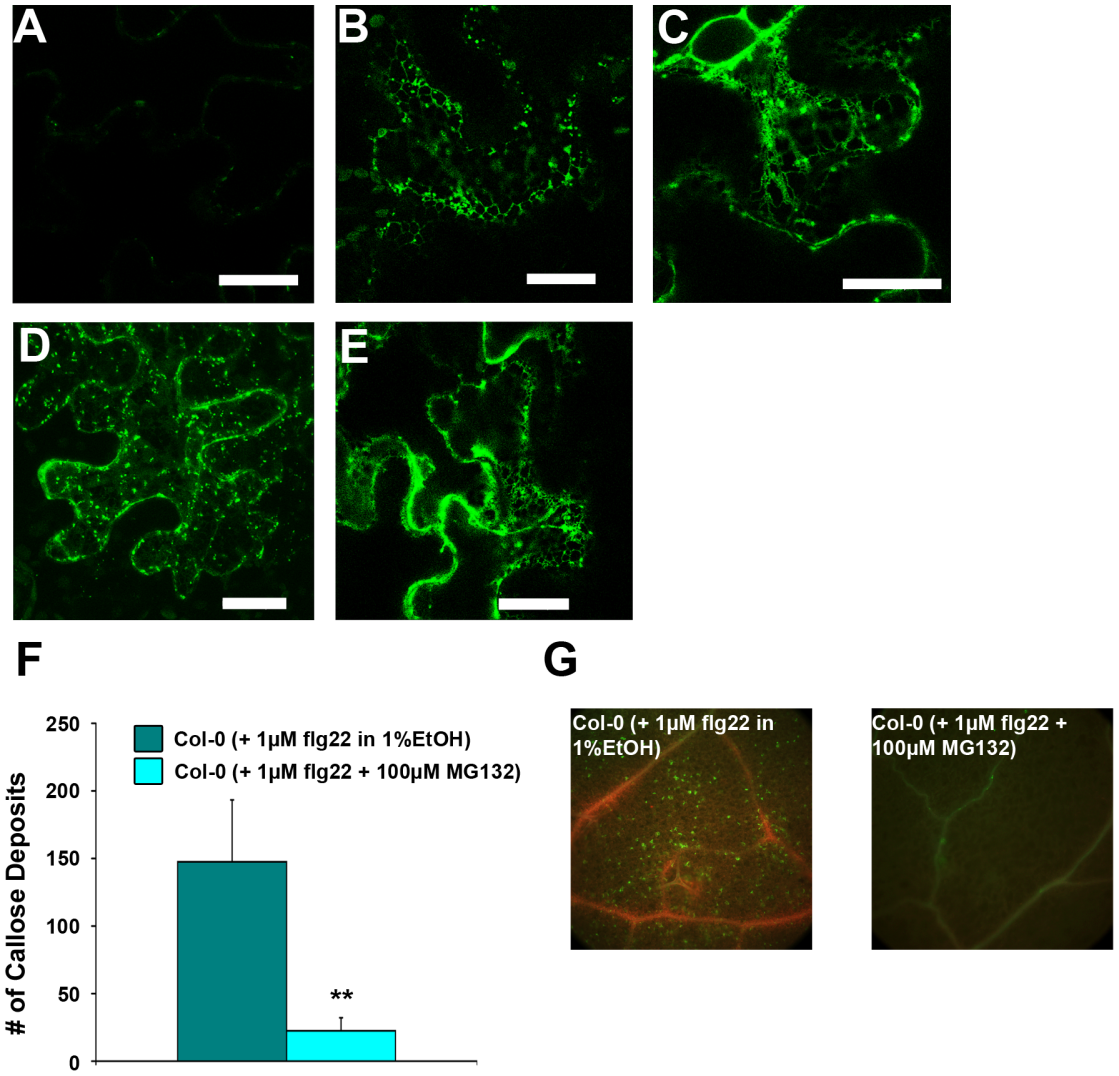
Inhibition of the proteasome by MG132 affects basal defence responses. MG132 treatment blocks secretion and leads to the accumulation of secreted green fluorescent protein (secGFP) within a cytosolic reticulum. Confocal images of *N. benthamiana* leaf epidermis cells transiently expressing (A) secGFP alone (1% EtOH) and (B)/(C) together with Sp2/XopJ. (D) secGFP- expressing leaf infiltrated with MG132 (300 µM) (E) or 100 µM MG132. Pictures in (D) and (E) were taken 2 h after infiltration. Bars = 20 µm. (F) Plants were co-infiltrated with 1 µM flg22+1% EtOH or 100 µM MG132. Leaf tissue was collected 6 hours after treatment and stained for callose. (G) Quantification of callose depositions per field of view. Data represent the mean SD (n = 4). Significant differences are indicated by asterisks (** P<0.01) and were calculated using Student's t-test.

As a consequence of a secretory block, coexpression of secGFP with Sp2 or XopJ-myc in leaves of *N. benthamiana* plants led to the expected increase in secGFP fluorescence, forming an intracellular ER-like pattern of fluorescence (Figure 11B and C). When plants transiently expressing secGFP were infiltrated with 100 µM MG132 and subjected to confocal microscopy one hour after the treatment detection of ER-like GFP fluorescence indicated that GFP secretion into the apoplast was impaired (Figure 11E). At a higher MG132 concentration (300 µM) fast moving punctuate structures became visible that could represent some sort of vesicles (Figure 11D).

Taken together, these data indicate that manipulation of the proteasome function by inhibition through MG132 affects protein secretion, which opens the possibility that XopJ acts on secretion in a similar manner. However, the experiments described here do not show that MG132 and XopJ necessarily act in the same way to inhibit secretion.

To further explore the effects of proteasome inhibition on basal defence responses, callose deposition was analyzed, after flg22 challenge in the presence or absence of MG132. Six hours after treatment with flg22 plants infiltrated with MG132 exhibited significantly reduced callose deposits (Figure 11F and G), indicating that the inhibition of the proteasome could interfere with cell-wall associated defence in Arabidopsis.

## Discussion

Type III effector proteins delivered into the host cell by plant-pathogenic bacteria collectively suppress host defence responses to promote virulence and pathogen spread. However, specific host cellular targets have been identified for only a few T3Es and the majority seems to interfere with defence signalling [2], [3]. We have previously shown that the Xcv T3E XopJ is able suppress protein secretion and callose deposition when transiently expressed in leaves of *N. benthamiana* or after inducible expression in Arabidopsis, respectively, and thus this effector apparently can interfere with cell wall-based basal defence [19].

In the present study we identified the 26S proteasome subunit RPT6 as a potential virulence target of XopJ in plant cells. We demonstrated that XopJ interacts with RPT6 from different plant species in yeast and *in planta*. Furthermore, wild type XopJ inhibits proteasome activity, reduces accumulation of SA, and attenuates the onset of necrosis and pathogen-induced senescence during infection of susceptible pepper plants with Xcv.

The 26S proteasome is an essential multicatalytic protease complex for the degradation of regulatory proteins that have been marked for destruction by ubiquitin (Ub). The ubiquitin/proteasome system (UPS) plays a central role in the degradation of short-lived and regulatory proteins important for a variety of cellular processes [21], [39]. It consists of two multisubunit protein complexes: the 20S proteolytic core protease (CP) and the 19S regulatory particle (RP) that is composed of 17 subunits. The CP functions as a nonspecific ATP and Ub-independent protease that forms a cylindrical structure composed of four heptameric rings while the RP caps one or both ends of the CP and confers ATP dependence and poly-Ub recognition to the proteasome. The RP is composed of a ring of six triple A (AAA^+^) ATPases (RPT1-6) that covers the opening to the CP and probably assists in target unfolding, the RP non ATPases (RPNs) 10 and 13 are Ub receptors, and RPN11 is a deubiquitylating enzyme (DUB) that helps to release bound Ub [21]. Arabidopsis mutants compromised in individual proteasome subunits often display severe developmental defects or even show embryo lethality, confirming the importance of the UPS for overall plant fitness [40].

During the past few years, a growing body of evidence has indicated that the UPS is not only implicated in crucial cellular survival mechanisms, but also plays a central role in plant defence during PTI as well as during ETI [38], [41]. For example, several E3 ligases have been shown to be required for the development of HR in Cf-9 mediated resistance in response to the fungal avirulence protein Avr9 in tobacco [42]. Furthermore, RING-finger E3 ubiquitin ligases in *Arabidopsis* are involved in RPM1- and RPS2-mediated elicitation of the HR [43] and *Arabidopsis* PUB17 (an U box E3 ligase) knockout plants are compromised in RPS4-mediated resistance against *Pseudomonas syringae* pv. *tomato* containing avirulence genes AvrB and AvrRPS4 [44]. The U-box E3 ligases PUB22, 23, and 24 have been demonstrated to negative regulate PTI responses in *Arabidopsis* [45]. In contrast, a number of bacterial T3Es have been shown to exploit the host cell UPS for instance by acting as E3 ligases, such as AvrPtoB [46], [47], or by otherwise promoting ubiquitination of target proteins in order to suppress plant defence, like HopM1 [48]. However, proteasomal subunits have yet not been identified as direct targets of T3Es in plants. Recently, it has been shown that *P. syringae* pv. *syringae* strain B728a secretes a small non-ribosomal peptide called syringolin A (SylA) which acts as an inhibitor of the host cell proteasome [24]. A SylA deficient strain caused reduced symptom development on susceptible bean plants suggesting that SylA contributes to bacterial virulence. Thus, the proteasome appears to represent a valid target for T3Es to promote bacterial virulence. Because of its central role in numerous regulatory pathways, inhibition of the proteasome by bacterial effector molecules can be expected to elicit pleiotropic responses and it is not obvious how the pathogen would benefit from these. The analysis of Arabidopsis mutants compromised in certain subunits of the proteasome suggests that the proteasome contributes to basal defence responses. An Arabidopsis *rpn1a* mutant displayed enhanced susceptibility toward virulent and avirulent *P. syringae* strains as well as to the biotrophic fungal pathogen *Golovinomyces cichoracearum* while no effect on virulence could be observed upon infection of mutant plants with *Botrytis cinerea*, a necrotroph [49]. This led to the suggestion that RPN1a is involved in resistance against biotrophic pathogens, but not necrotrophic pathogens. From a number of additional Arabidopsis proteasome subunit mutants tested only those affected in RPT2a and RPN8a function fully suppressed *edr2*-mediated powdery mildew resistance indicating that the different proteasome subunits might have distinct roles in mediating plant defence responses [49]. *RPT6* knock-out alleles in Arabidopsis have thus far not been described; however, our own experiments show that suppression of *RPT6* expression by VIGS in pepper is lethal to the plants. This precludes a genetic analysis of RPT6 function in this system but it implies that RPT6 is essential for proper proteasome function and thus constitutes a valid target for T3Es to interfere with proteasome activity. In contrast to a previous study [17] we could show that a Xcv *xop*J deletion mutant was slightly but significantly reduced in bacterial growth in a compatible interaction with pepper plants. Thus, the question arises as to how a XopJ-mediated inhibition of the proteasome contributes to bacterial virulence. Measurement of overall proteasome activity in Xcv infected pepper leaves revealed a significant induction in proteasome activity that was even higher upon infection with an Xcv Δ*xop*J deletion strain. On the one hand this indicates that XopJ indeed contributes to suppression of proteasome activity during infection, confirming the findings from transient expression of XopJ, while on the other hand infection with virulent Xcv *per se* appears to induce the proteasome most likely as a consequence of slightly elevated SA levels in the course of induced defence. This is further corroborated by the fact that infection of pepper with a Xcv Δ*hrpF* mutant, which lacks a functional T3SS and thus is not able to suppress basal defence responses, causes induction of the proteasome comparable to that seen upon Xcv Δ*xop*J infection. Thus, induction of proteasome activity might be a component of basal defence. However, it could also be possible that Xcv translocates T3Es that require and thus induce proteasome activity for their virulence function as it has been shown for effectors from a range of other bacterial pathogens [50]. A recent publication suggests that XopL of Xcv exhibits E3 ubiquitin ligase activity *in planta* and is able to subvert plant immunity [51]. Hence, XopL virulence function would rely on a functional proteasome. This apparent contradiction can be resolved if UPS-related T3Es would act spatially separated from XopJ. The *in planta* BiFC analysis indicates that the interaction between XopJ and RPT6 occurs at or close to the PM. The proteasome is assumed to be distributed between the cytosol and the nucleus of the cell; however, proteomics studies imply that RPT2 can be myristoylated [52], [53] and thus could target a subset of 26S particles to the PM where they might serve a specialized function. Along that line, it has been shown that a RPT2a G2A mutant was able to rescue most but not all of the phenotypic functions of RPT2a [54].

Xcv can be considered as a hemibiotrophic pathogen that exhibits characteristics of both biotrophs and necrotrophs, depending on the stages of its life cycle. In the early stages of infection, Xcv requires living host tissue to thrive and multiply. Thus, host programmed cell death has been suggested to constitute an effective way to mount an efficient defence response towards a pathogen in the biotrophic phase while the bacterium would need to repress this reaction in order to allow disease progression. Leaves infected with an Xcv Δ*xop*J deletion strain developed necrotic lesions as early as 3 dpi while Xcv wild type infected leaves appeared asymptotic at this time point. This suggests that XopJ acts to prevent host cell death during the biotrophic phase of infection. Given the fact that an Xcv Δ*xop*J deletion strain is slightly impaired in bacterial multiplication during infection of susceptible pepper plants it seems that the XopJ mediated delay in the development of tissue necrosis is required for full pathogen growth in pepper. A similar role has been proposed for XopD during the compatible interaction of Xcv with tomato [28]. XopD alters the kinetics of leaf chlorosis and necrosis without affecting the number or rate of appearance of necrotic lesions and it furthermore acts as a tolerance factor to increase the ability of the host to cope with bacterial colonization. Interestingly, it appears that XopD is not required for full virulence of Xcv on pepper and it has been suggested that another T3E could play a functionally redundant role in pepper and thus might mask XopD action [28]. Future studies using a *xopD/xop*J double mutant could shed further light on a functionally redundant role of these two T3Es.

In tomato, there are two stages of disease symptom development during infection with Xcv [26], [55]. The primary response consists of localized lesions, which is followed by a secondary phase of chlorosis and necrosis spreading out from the primary sites of infection. It has been shown that the infection of tomato with virulent Xcv was associated with a substantial increase in SA levels. The analysis of transgenic tomato plants deficient in the accumulation of SA indicates that this secondary phase of Xcv-induced disease is SA dependent as these plants did not develop tissue necrosis [26]. The infection experiments of pepper with a XopJ deficient Xcv strain presented in this study show that XopJ is necessary to prevent SA accumulation and SA associated signalling during infection and the suppression of tissue necrosis by XopJ is likely a consequence of reduced SA signalling. The fact that XopJ alters the abundance of SA marker genes as well as of genes involved in development of leaf senescence provides further evidence for a role of XopJ in the suppression of SA-dependent host responses. Due to its role in triggering host programmed cell death SA is considered to be a central regulator of defence against biotrophic and hemi-biotrophic pathogens [56]. However, Arabidopsis basal defence responses such as bacterial-induced stomatal closure and callose deposition at the cell wall are at least in part also SA dependent [57], [58]. Consequently, several T3Es from virulent bacteria target the SA pathway to promote pathogenesis. For example, the *P. syringae* effector HopI1 localizes to chloroplasts where it suppresses SA accumulation [59]. The defense-suppressive activity of HopI1 depends on its interaction with the plant stress chaperone HEAT SHOCK PROTEIN 70 (HSP70), which is thought to possess a defence-promoting function [60]. In addition, *hopPtoM* and *avrE* genes of *P. syringae* were found to encode suppressors of these SA-dependent basal defense responses, such as callose deposition [58]. Kim et al. [28] could show that during infection of tomato with Xcv XopD functions as a transcriptional repressor, resulting in the suppression of SA-induced defence responses that otherwise would limit Xcv growth. However, in a more recent study the same authors could show that XopD affects ethylene signalling and the suppression of SA responses might rather be a secondary effect [61]. Thus far none of the T3Es affecting SA signalling has been functionally associated with the proteasome and there is still a possibility that inhibition of SA responses by XopJ is an indirect effect through interference with upstream processes. Strikingly, the SylA peptide secreted by *P. syringae* pv. *syringae* strain B728a was shown to inhibit stomatal innate immunity in bean and Arabidopsis through inhibition of the proteasome [62]. Further analysis revealed that stomatal closure in response to infection was dependent on an intact SA signalling pathway. Thus, it appears likely that XopJ suppresses SA signalling and the development of tissue necrosis by inhibition of the proteasome through its interaction with RPT6. Additional support for a role of the proteasome in SA mediated defence response is provided by the finding that Arabidopsis *rpn1a*, as well as *rpt2a* and *rpn8a* mutants accumulated significant lower SA levels than wild type upon infection with virulent Pto DC3000 [49]. It is currently unclear how inhibition of the proteasome by XopJ could affect SA accumulation and signalling. The most parsimonious explanation is that XopJ would inhibit the proteasomal turnover of a negative regulator of SA synthesis or signalling. The master regulator of SA responses in plants NPR1 (*n*onepxressor of *p*athogenesis-*r*elated (PR) genes), a transcriptional co-activator, must be turned over in its phosphorylated form by the proteasome to activate SA responsive genes [63]. Inhibition of the proteasome by XopJ could prevent NPR1 turnover and thus downstream SA responses. However, our results indicate that XopJ interacts with RPT6 close to or at the PM while proteasomal turnover of NPR1 occurs within the nucleus [63]. On the other hand it is currently unknown whether XopJ only inhibits a subpopulation of the proteasome at the PM or whether proteasome complexes in other cellular locations would also be affected. Alternatively, other components of SA signalling could be dependent on proteasomal turnover at the PM. Recently it could be shown that the PM localized RING E3 ubiquitin ligase CaRING1 is required for SA accumulation and induction of SA responsive marker genes in pepper [64]. The observation that the loss of XopJ could be mimicked by external SA application onto wild type Xcv infected pepper leaves might argue for an intact SA signalling which is just not triggered due to too low internal SA levels. In this scenario XopJ would rather interfere with SA synthesis than with signalling. However, VIGS of *NPR1* expression in pepper abrogates early tissue necrosis upon infection with Xcv Δ*xop*J demonstrating the necessity of a functional SA signalling pathway for this type of host cell death to occur. Although this observation does not necessarily imply that proteasome suppression by XopJ directly interferes with NPR1 function it strongly suggests that XopJ acts to inhibit SA mediated defence responses.

Our data also indicate that SA induces RPT6 transcription as well as overall proteasome activity suggesting a positive feedback mechanism to amplify the proteasome dependent induction of SA synthesis or signalling. In Arabidopsis, SA treatment led to the induction of RPN1a expression [49] and spraying plants with benzothidiazole (BTH) to induce SA signalling results in increased activity of the proteasome [65]. Pepper plants with reduced expression of NPR1 did not show an increase in proteasome activity upon infection with either Xcv wild type or the Xcv Δ*xop*J mutant indicating that the regulation of proteasome activity during defence is at least partly mediated through NPR1. This is in line with the observation that induction of proteasome activity upon BTH treatment is abolished in an Arabidopsis *npr1* knock-out mutant [65]. It is currently not known whether the SA-mediated increase in proteasome activity in pepper is solely due to an increase in gene expression or whether post-translational mechanisms might also contribute to activation.

We have previously shown that XopJ inhibits cell wall – associated defence responses such as protein secretion and callose deposition [19]. We could mimic the inhibitory effect of XopJ on secretion of a GFP reporter by applying the proteasome inhibitor MG132 to leaves. It is currently unknown whether proteasome activity is directly required for secretion or whether proteasome inhibition acts indirectly for instance by interference with SA which has been shown to control expression of secretory pathway genes [66]. Treatment of Arabidopsis leaves with MG132 also prevented callose deposition upon challenge with flg22 indicating that proper proteasome function is also required for this type of defence response. These results open the possibility that the previously observed effects of XopJ on protein secretion and callose deposition could well be mediated by the ability of XopJ to inhibit the proteasome.

How then does XopJ act mechanistically to inhibit the proteasome? Several members of the YopJ-like effector family posses acetyltransferase activity [10], [15], [16], [67]. Although the trans-acetylation substrate for these T3Es has not been identified in all cases they have been shown to autoacetylate most likely on a conserved lysine residue [15], [16]. Although this lysine residue is also present in XopJ [K300; 16] we could neither demonstrate autoacetylation of XopJ nor acetyltransferase activity using recombinant *E. coli* produced XopJ in the presence of ^14^C-acetyl CoA with RTP6 as a *bona fide* substrate (data not shown). It has recently been shown that HopZ1a from *P. syringae* requires phytic acid as a cofactor for its full acetyltransferase activity [15]. Phytic acid has been previously shown to activate the acetyltransferase activities of YopJ and AvrA, both highly divergent homologs of HopZ1a from animal pathogens [67] and thus might represent a general activator for these effectors. However, in case of XopJ also the addition of phytic acid to the assay did not lead to any detectable acetyltransferase activity (data not shown). HopZ1a as well as AvrBsT from *Xcv* have weak cysteine protease activities *in vitro* [9], [68]. Whether XopJ could act as a protease to mediate destabilization of RPT6 will be subject of future studies.

While in yeast a C235A mutation of XopJ abolished its ability to interact with RPT6 the XopJ(C235A) mutant is still able to interact with RPT6 *in planta* although it is no longer able to inhibit the proteasome. This discrepancy in binding of the XopJ(C235A) mutant to RPT6 in the two experimental systems is currently unresolved but could reflect differences in sensitivity between the two methods. In addition, we currently cannot exclude the C235A mutation affects protein level or other properties of the protein in yeast which in turn could also affect interaction with RPT6. Taken together, however, our results strongly argue for an enzymatic activity of XopJ of whatever kind this might be and it appears likely that the effector requires an as yet unknown host cell factor or a host cell mediated posttranslational modification for activation.

In conclusion, we have shown that XopJ interacts with the proteasomal subunit RPT6 to inhibit proteasome activity. XopJ represses SA-mediated defence responses and counteracts development of tissue damage during a compatible interaction of Xcv with pepper providing a growth advantage to the bacterium at late stages of infection. Prolonged host cell viability through inhibition of the proteasome is likely to enhance nutrient availability and could also support pathogen spread from the initial site of infection. Future studies will have to reveal the biochemical activity of XopJ and further establish the connection of proteasome activity with SA signalling on the molecular level.

## Materials and Methods

### Plant material and growth conditions

Pepper (*Capsicum annuum* cv. Early Cal Wonder (ECW)) and tobacco plants (*Nicotiana benthamiana*) were grown in soil in a greenhouse with daily watering, and subjected to a 16 h light :8 h dark cycle (25°C : 21°C) at 300 µmol m^−2^ s^−1^ light and 75% relative humidity.

### Infection of pepper plants

Xcv infections and bacterial growth assays were performed as described previously [69].

### Yeast two-hybrid analysis

Yeast two-hybrid techniques were performed according to the yeast protocols handbook and the Matchmaker GAL4 Two-hybrid System 3 manual (both Clontech, Heidelberg, Germany) using the yeast reporter strains AH109 and Y187. The entire XopJ coding region was amplified by PCR using the primers listed in Table S1 and inserted in the pGBT-9 vector generating a fusion between the GAL4 DNA-binding domain (BD). The yeast strain Y187 carrying the BD-XopJ construct was mated with AH109 cells pre-transformed with either a two-hybrid library from *Arabidopsis* inflorescence [70] (kindly provided by the Arabidopsis Biological Resource Center) or with a library derived from tobacco (*Nicotiana tabacum*) source leaves [71]. Diploid cells were selected on medium lacking Leu, Trp, and His supplemented with 4 mM 3-aminotriazole. Cells growing on selective medium were further tested for activity of the *lacZ* reportergene using filter lift assays. Library plasmids from *his3/lacZ* positive clones were isolated from yeast cells and transformed into *E. coli* before sequencing of the cDNA inserts. Direct interaction of two proteins was investigated by cotransformation of the respective plasmids in the yeast strain AH109, followed by selection of transformants on medium lacking Leu and Trp at 30°C for 3 days and subsequent transfer to medium lacking Leu, Trp and His for growth selection and *lacZ* activity testing of interacting clones.

For the generation of the AtRPT6b, ScRPT6 and CaRPT6 activation domain fusions the respective coding region was amplified by PCR using the primers listed in Table S1, inserted into the vector pGAD424 (Clontech) and sequence verified.

### Site directed mutagenesis

Site directed mutagenesis of XopJ constructs was carried out using the Quick-change site directed mutagenesis kit (Stratagene, Heidelberg, Germany) employing primers listed in Table S1 online. All base changes were verified by sequencing.

### Plasmid construction for transient expression experiments

Construction of binary vectors expressing XopJ and its mutant variants XopJG2A and C235A was described previously [19]. The *Nt*RPT6-GFP construct was assembled by amplifying the entire coding region from tobacco cDNA using the primers listed in Table S1. The resulting PCR fragment was inserted in the pENTR-D/TOPO (Invitrogen) and subsequently recombined into pK7WGF2 [72] using L/R-Clonase (Invitrogen).

### BiFC assay

Entry clones of XopJ and *Nt*RPT6 comprising the entire coding region of each cDNA were used in a L/R-reaction with a Gateway-System (Invitrogen, Karlsruhe, Germany) compatible version of the BiFC vectors pUC-SPYNE and pUC-SPYCE, respectively [73]. Constructs were delivered into leaf cells of tobacco by particle bombardment using a Bio-Rad PDS – 1000 He particle delivery system according to the manufacturer's instructions. The BiFC-induced fluorescence was detected by confocal laser scanning microscopy on a Leica TCS SP5II after 24 h of incubation at 22°C in the dark.

### Agroinfiltration

For infiltration of *N. benthamiana* leaves, *A. tumefaciens* C58C1 was infiltrated into the abaxial air space of 4- to 6-week-old plants, using a needleless 2-ml syringe. Agrobacteria were cultivated overnight at 28°C in the presence of appropriate antibiotics. The cultures were harvested by centrifugation, and the pellet was resuspended in sterile water to a final optical density at (OD_600_) of 1.0. The cells were used for the infiltration directly after resuspension. Infiltrated plants were further cultivated in the greenhouse daily watering, and subjected to a 16 h light: 8 h dark cycle (25°C : 21°C) at 300 µmol m^−2^ s^−1^ light and 75% relative humidity.

### Western blotting

Leaf material was homogenized in sodium-dodecyl sulphate-polyacrylamide gel electrophoresis (SDS-PAGE) loading buffer (100 mM Tris-HCl, pH 6.8; 9% β-mercapto ethanol, 40% glycerol, 0.0005% bromophenol blue, 4% SDS) and, after heating for 10 min at 95°C, subjected to gelectrophoresis. Separated proteins were transferred onto nitrocellulose membrane (Porablot, Machery und Nagel, Düren, Germany). Proteins were detected by either an anti-HA antibody (Sigma), anti-myc antibody (Santa Cruz Biotechnology), anti-GFP antibody (Roche), or anti-ubiquitin antibody (Agrisera) via chemiluminescence (GE Healthcare).

### Generation of Xcv ΔxopJ

To generate deletions of *xop*J, a fragment ranging from position −67 to +147 relative to the ATG and another fragment comprising −96 to +74 relative to the stopp codon of the XopJ coding region were amplified from genomic DNA of *Xcv* 85-10 by PCR using oligonucleotides harboring appropriate restriction sites (Table S1). Both fragments were fused by PCR resulting in an internal deletion fragment. The fragment was subsequently cloned into suicide vector pOK1 [74] using BamHI and SalI restriction sites. The resulting constructs were conjugated into *Xcv* strain 85-10, and mutants were selected by PCR.

### Measurement of proteasome activity

Proteasome activity in crude plant extracts was determined spectrofluorometrically using the fluorogenic substrate suc-LLVY-NH-AMC (Sigma) according to Reinheckel et al. [75]. In brief, four leaf discs with a diameter of 0.7 cm each were harvested and frozen in liquid nitrogen. The leaf material was ground in 200 µl extraction buffer [50 mM HEPES-KOH, pH 7.2, 2 mM ATP, 2 mM DTT, 250 mM sucrose]. After centrifugation the protein concentration of the supernatant was adjusted to 1 mg/ml with extraction buffer. 50 µg of total protein was mixed with 220 µl proteolysis buffer [100 mM HEPES-KOH, pH 7,8, 5 mM MgCl2, 10 mM KCl, 2 mM ATP]. The reaction was started after 5 min at 30°C by addition 0,2 mM suc-LLVY-AMC. Released amino-methyl-coumarin (AMC) was measured every two minutes between t_0_ and t_120_ min using a fluorescence spectrophotometer (FLX800, BioTek), with an excitation wavelength of 360 nm and an emission wavelength of 460 nm. Proteasome activity was calculated from the linear slope of the emission curve and is expressed as fluorescence units per minute (RFU min^−1^) or in percentage relative to controls, respectively.

### Protein extraction and GFP-pull down in *N. benthamiana*

GFP-pull down assays were carried out according to Schwessinger et al. [76] with slight modifications. Approximately 1 g of leaf material was ground to fine powder in liquid nitrogen and 5 ml extraction buffer [50 mM Tris-HCl pH 7.5; 150 mM NaCl; 10% glycerol; 10 mM DTT; 10 mM EDTA; 1 mM NaF; 1 mM Na2MoO4.2H2O; 1% (w/v) PVPP; 1% (v/v) P9599 protease inhibitor cocktail (Sigma); 1% (v/v) NP-40] added. Samples were cleared by centrifugation at 16.000×g for 15 min at 4°C and adjusted to 2 mg/ml total protein concentration. Immunoprecipitation was performed on 1.5 ml total protein by adding 20 µl GFPTrap-M beads (Chromotek) and incubation at 4°C for 3–4 h. Beads were washed 4 times with TBS containing 0.5% (v/v) NP-40, immunoprecipitates eluted with 30 µl 2× SDS loading buffer and heating at 70°C for 10 min.

### Ion leakage measurements

For electrolyte leakage experiments, triplicates of 1.76 cm^2^ infected leaf material were taken at indicated time points. Leaf discs were placed on the bottom of a 15 ml tube. 8 ml of deionized water was added to each tube. After 24 h of incubation in a rotary shaker at 4°C, conductivity was determined with a conductometer. To measure maximum conductivity of the entire sample, conductivity was determined after boiling the samples for 30 min [77].

### Trypan blue staining

To visualize dying cells, leaves were detached and submerged in lactophenol-trypan blue solution (0.03% trypan blue, 33% [w/v] lactic acid, 33% water-saturated phenol, and 33% glycerol). Samples were incubated at 99°C for 1 min followed by incubation at room temperature for 24 h, washed in chloral hydrate solution (2.5 g mL^−1^) to reduce background staining, and photographed using a Leica MZLIII stereomicroscope (Leica Microsystems).

### RNA extraction and expression analysis

Total RNA was isolated from leaf material and then treated with RNAse-free DNase (Fermentas) to degrade any remaining DNA. First strand cDNA synthesis was performed from 2 µg of total RNA using a random hexamer using Revert-Aid reverse transcriptase (Fermentas). For quantitative realtime RT-PCR, the cDNAs were amplified using Brilliant II SYBR Green QPCR Mastermix (Stratagene) in an MX3000P real-time PCR instrument (Stratagene). PCR was optimized, and reactions were performed in triplicate. The transcript level was standardized based on cDNA amplification of *Actin* as a reference. Fold induction values of target genes were calculated with the ΔΔCP equation according to Pfaffl [78]. Statistical analysis was performed using a two tailed Student's t-test. Primers used for RT-PCR and quantitative real-time PCR, respectively, are listed in Table S1.

### Determination of SA and SA glucoside contents

Free SA and SA glucoside were extracted and analyzed as described [79].

### Callose assay

Six-week-old *Arabidopsis* Col-0 plants were infiltrated with a mixture of 1 µM flg22+1%EtOH or 100 µM MG132 if not otherwise stated. Leaf tissue was harvested 6 hpi and cleared of pigments by treatment with Lactophenol (95% EtOH: 5% Lactophenol). After staining of leaf material with aniline blue solution (0.01% aniline blue in 0.15M K_2_HPO_4_, pH 9.5), leaves were examined with a Leica DMR microscope. The number of callose deposits was determined on four microscopic views taken from four independent leaves. The callose assays reported here were performed two times with similar results.

### VIGS

VIGS was performed as described previously [69]. Briefly, Agrobacterium strains with the pTRV1 vector and with pTRV2-GFPsil, PYL279-RPT6 and pTRV2-NPR1 [80] (OD 600 = 1.0) were mixed in a 1∶1 ratio, respectively, and the mixture was infiltrated into cotyledons of 2-week-old pepper plants using a 1-mL sterile syringe without a needle. The Agrobacterium-inoculated pepper plants were grown in the green house at 20°C/16°C in the dark for 56 h with 45% relative humidity, and then transferred to a 16 h light :8 h dark cycle (25°C : 21°C) at 300 µmol m^−2^ s^−1^ light and 75% relative humidity.

### *In vitro* pull-down

Recombinant proteins from *Escherichia coli* lysates were immobilized on amylose resins (New England Biolabs), incubated for 1 h at 4°C with purified GST-RPT6, eluted, and analyzed by immunoblotting using either anti-GST antibody (Sigma) or anti-MBP antibody (NEB).

### MG132 treatment

For callose deposition, plants were co-infiltrated with 1 µM flg22+1% EtOH or 100 µM MG132. For the analysis of secretion, 100 or 300 µM MG132 or 1% EtOH was infiltrated to plants transiently expressing secGFP 44 hpi. At 46 hpi plants were analysed under the CLSM. For MG132 treatment of pepper leaves, plants were first infiltrated with Xcv strains. Inoculated areas were then infiltrated with 100 µM MG132 or 1% EtOH at 2 dpi. Leaves were photographed 3 dpi.

### Accession numbers

Sequence data from this article can be found in the Arabidopsis Genome Initiative or GenBank/EMBL databases under following accession numbers: At5g19990 (AtRPT6a); At5g20000 (AtRPT6b); JX965405 (NtRPT6); JX965404 (CaRPT6); EHN02423.1 (ScRPT6). Accession numbers of proteins used for sequence alignments can be found in the legend of the respective figures.

## Supporting Information

Figure S1 **Protein sequence alignment of RPT6 from different species.** (**A**) The alignment was generated using CLUSTALW2 with default parameters and BoxShade 3.21. Positions of identical and similar sequences are boxed in black and grey, respectively. The following sequences were used to build the alignment: *Mus musculus* NP032976; *Rattus norvegicus* BAA22935; *Homo sapiens 1* NP_002796.4; *Homo sapiens 2* NP_001186092.1; *Drosophila melanogaster* NP_608447.1; *Arabidopsis thaliana* isoform a At5g19990; *Arabidopsis thaliana* isoform b At5g20000; *Nicotiana tabacum* JX965405; *Capsicum annuum* JX965404, *Oryza sativa* NP_001046248.1; *Saccharomyces cerevisiae* EHN02423.1 (B) Similarity matrix of RPT6 sequences from different species. Degree of similarity is given in percent.
(PDF)

Figure S2 **Control experiments for BiFC assays.** YFP confocal microscopy images show tobacco leaf epidermal cells transiently expressing constructs encoding the fusion proteins indicated. Merge indicates an overlay of the YFP and chlorophyll autofluorescence images. Each image is the representative of at least two experiments. Bars = 10 µm if not otherwise indicated.
(PDF)

Figure S3 **Proteasome inhibition is specific for XopJ.** (**A**) XopJ protein along with an empty vector (EV) control and XopB-myc were transiently expressed in leaves of *N. benthamiana* using Agro-infiltration. After 48 h, relative proteasome activity in total protein extracts was determined by monitoring the breakdown of the fluorogenic peptide Suc-LLVY-NH-AMC at 30°C in a fluorescence spectrophotometer. The empty vector (EV) control was set to 100%. Data represent the mean + SD (n = 3). (**B**) Immunodetection of transiently expressed XopJ and XopB in the same leaves that were used for proteasome activity measurements. After immunodetection of proteins the membrane was stained with amido black to control for equal protein loading.
(PDF)

Figure S4 **Phenotype of Xcv infected pepper leaves 5 dpi.** Xcv (vector), Xcv *Δxop*J (vector), Xcv *Δxop*J (XopJ-HA), XopJ (G2A-HA) and (C235A-HA) were inoculated at a bacterial density of 2×10^8^ cfu ml^−1^ into leaves of pepper ECW plants. Pictures were taken at 5 dpi.
(PDF)

Figure S5 **XopJ delivered by a complemented Xcv** ***Δxop***J** (XopJ-HA) is able to suppress the phenotype of the Xcv Δ** ***xop***J** strain in a mixed inoculum experiment.** Leaves of pepper ECW plants were first inoculated with Xcv Δ*xop*J (XopJ-HA) at a density of 2×10^8^ cfu ml^−1^. After 3 h the same leaf region was inoculated with Xcv Δ*xop*J bacteria of the same density. The phenotype of the infected leaf was recorded 3 dpi.
(PDF)

Figure S6 **SA treatment induces tissue necrosis in Xcv wild type infected pepper leaves.** Xcv wild type and Xcv *Δxop*J were inoculated at a bacterial density of 2×10^8^ cfu ml^−1^ into leaves of pepper ECW plants. 2 dpi Xcv wild type infected leaves were sprayed with 5 mM SA (middle) and the phenotype development was documented 3 dpi. Xcv wild type infected leaf sprayed with water (left) served as a control. For comparison phenotype development 3 dpi of aXcv *Δxop*J infected leaf is shown (right).
(PDF)

Figure S7 **RPT6 gene expression during compatible Xcv-pepper interaction.** Total RNA was isolated from pepper leaves infiltrated with 2×10^8^ cfu/mL of Xcv, Xcv *Δxop*J and 1 mM MgCl_2_. Quantitative real-time RT-PCR was performed for *CaRPT6* three dpi. *Actin* expression was used to normalize the expression value in each sample, and relative expression values were determined against the average value of the sample infected with 1 mM MgCl_2_. Leaf material from 4 independent pepper plants was pooled and analyzed in triplicates. Data represent the mean SD. Significant differences were calculated using Student's t-test and are indicated by: *, P<0.05; **, P<0.01.
(PDF)

Figure S8 **XopJ does not inhibit flg22-triggered MAPK signalling in Arabidopsis.** (A) flg22 mediated MAPK activation un-induced alc-XopJ plant in comparison to those induced with ethanol. Leaves were treated with 1 µM flg22, and MAPK activation was analyzed at the indicated time points. Upper panel: protein extracts (15 µg per lane) were subjected to western-blot analysis using the phospho-p44/p42 MAPK antibody. Arrowheads indicate the positions of MPK3 and MPK6. Lower panel: equal protein level of MPK3 and MPK6 in each lane was verified using an anti-MPK6 antibody. (B) Proteasome activity before and after treatment of alc-XopJ plants with 1% ethanol.
(PDF)

Figure S9 **Virus-induced gene silencing of RPT6 in ECW pepper plants.** (A) qRT-PCR analysis of RPT6 mRNA level in RPT6 silenced pepper plants. The log_2_ value is given, where −3.32 corresponds to a 10-fold down-regulation in the VIGS – RPT6 plants compared with the control (B) Phenotype of RPT6 - VIGS plants in comparison to the pTRV2-GFPsil control. Picture was taken 21 dpi.
(PDF)

Table S1 **Oligonucleotides used in this study.**
(PDF)
