# Supplementary material for: The Xanthomonas campestris Type III Effector XopJ Targets the Host Cell Proteasome to Suppress Salicylic-Acid Mediated Plant Defence
Source: PLoS Pathog. 2013 Jun 13;9(6):e1003427. doi: 10.1371/journal.ppat.1003427 (PMC3681735; doi:10.1371/journal.ppat.1003427)
Supplement: Figure S1 — Protein sequence alignment of RPT6 from different species. (A) The alignment was generated using CLUSTALW2 with default parameters and BoxShade 3.21. Positions of identical and similar sequences are boxed in black and grey, respectively. The following sequences were used to build the alignment: Mus musculus NP032976; Rattus norvegicus BAA22935; Homo sapiens 1 NP_002796.4; Homo sapiens 2 NP_001186092.1; Drosophila melanogaster NP_608447.1; Arabidopsis thaliana isoform a At5g19990; Arabidopsis thaliana isoform b At5g20000; Nicotiana tabacum JX965405; Capsicum annuum JX965404, Oryza sativa NP_001046248.1; Saccharomyces cerevisiae EHN02423.1 (B) Similarity matrix of RPT6 sequences from different species. Degree of similarity is given in percent. (PDF) [file ppat.1003427.s001.pdf]

Supplementary data to Üstün, S., Bartetzko, V., and Börnke, F.

Figure S1

A

```
M. musculus      1 -MALDGPEQMELEEG-----------------KAGSGLRQYYLSKIEELQLIVNDKSQNL
R. norvegicus    1 -MALDGPEQMELEEG-----------------KAGSGLRQYYLSKIEELQLIVNDKSQNL
H. sapiens 1     1 -MALDGPEQMELEEG-----------------KAGSGLRQYYLSKIEELQLIVNDKSQNL
H. sapiens 2     1 ---------MELEEG-----------------KAGSGLRQYYLSKIEELQLIVNDKSQNL
D. melanogaster  1 ---MTVTNRMEIESAY----------------HKGEGFRSYYIQKIEELQLVVAEKHQNL
A. thaliana a    1 -MAAVGVDSRRPETAM--EETCNVKG---AAAKQGEGLKQYYLQHIHELQRQLRQKTNNL
A. thaliana b    1 -MAAVGVEARPPVTAM--EETCNVKG---AAAKQGEGLNKYYLQHLDELQRLQREKSYNL
N. tabacum       1 -MASADVEKSRREIER--EESCSAK-----ATKQGEGLRQYYMQHIHDLQLQVRQKTHNL
C. annuum        1 -MASVDVGRSRMEAER--EEICSAK-----ATKQGEGLRQYYMQHIHDLQLQVRHKTHNL
O. sativa        1 -MATVAMDISKPPPAAGGDEAAAAKGRGGAGGGGGEGLRQYYLQHIHDLQLQIRQKTHNL
S. cerevisiae    1 MTAAVTSSNIVLET-------------------HESGIKPYFEQKIQETELKIRSKTENV

M. musculus     43 RRLQAQRNELNAKVRLLREELQLLQEQGSYVGEVVRAMDKKKVLVKVHPEGKFVVDVDKN
R. norvegicus   43 RRLQAQRNELNAKVRLLREELQLLQEQGSYVGEVVRAMDKKKVLVKVHPEGKFVVDVDKN
H. sapiens 1    43 RRLQAQRNELNAKVRLLREELQLLQEQGSYVGEVVRAMDKKKVLVKVHPEGKFVVDVDKN
H. sapiens 2    35 RRLQAQRNELNAKVRLLREELQLLQEQGSYVGEVVRAMDKKKVLVKVHPEGKFVVDVDKN
D. melanogaster 42 RRLQAQRNELNAKVRMLREELQLLQEQGSYVGEVVKPMDKKKVLVKVHPEGKFVVDLDKN
A. thaliana a   55 NRLEAQRNELNSRVRMLREELQLLQEPGSYVGEVVKVMGKNKVLVKVHPEGKYVVDIDKS
A. thaliana b   55 NRLEAQRNELNSRVRMLREELQLLQEPGSYVGEVVKVMGKNKVLVKVHPEGKYVVDIDKS
N. tabacum      53 NRLEAQRNELNSKVRMLKEELQLLQEPGSYVGEVVKVMGKSKVLVKVHPEGKYVVDIDKS
C. annuum       53 NRLEAQRNELNSKVRMLKEELQLLQEPGSYVGEVVKVMGKSKVLVKVHPEGKYVVDIDKT
O. sativa       60 NRLEAQRNDLNSRVRMLREELQLLQEPGSYVGEVVKVMGKSKVLVKVHPEGKYVVDIDKS
S. cerevisiae   42 RRLEAQRNALNDKVRFIKDELRLLQEPGSYVGEVIKIVSDKKVLVKVQPEGKYIVDVAKD

M. musculus    103 IDINDVTPNCRVALRNDSYTLHKILPNKVDPLVSLMMVEKVPDSTYEMIGGLDKQIKEIK
R. norvegicus  103 IDINDVTPNCRVALRNDSYTLHKILPNKVDPLVSLMMVEKVPDSTYEMIGGLDKQIKEIK
H. sapiens 1   103 IDINDVTPNCRVALRNDSYTLHKILPNKVDPLVSLMMVEKVPDSTYEMIGGLDKQIKEIK
H. sapiens 2    95 IDINDVTPNCRVALRNDSYTLHKILPNKVDPLVSLMMVEKVPDSTYEMIGGLDKQIKEIK
D. melanogaster102 IDINDVTPNCRVALRNESYTLHKILPNKVDPLVSLMMVEKVPDSTYEMVGGLDKQIKEIK
A. thaliana a  115 IDITKITPSTRVALRNDSYVLHLVLPSKVDPLVNLMKVEKVPDSTYDMIGGLDQQIKEIK
A. thaliana b  115 IDITKLTPSTRVALRNDSYVLHLVLPSKVDPLVNLMKVEKVPDSTYDMIGGLDQQIKEIK
N. tabacum     113 IDITKITPSTRVALRNDSYVLHLILPSKVDPLVNLMKVEKVPDSTYDMIGGLDQQIKEIK
C. annuum      113 IDITKITPSTRVALRNDSYVLHLILPSKVDPLVNLMKVEKVPDSTYDMIGGLDQQIKEIK
O. sativa      120 IDITKITPSTRVALRNDSYMLHLILPSKVDPLVNLMKVEKVPDSTYDMIGGLDQQIKEIK
S. cerevisiae  102 INVKDLKASQRVCLRSDSYMLHKVLENKADPLVSLMMVEKVPDSTYDMVGGLTKQIKEIK

M. musculus    163 EVIELPVKHPELFEALGIAQPKGVLLYGPPGTGKTLLARAVAHHTDCTFIRVSGSELVQK
R. norvegicus  163 EVIELPVKHPELFEALGIAQPKGVLLYGPPGTGKTLLARAVAHHTDCTFIRVSGSELVQK
H. sapiens 1   163 EVIELPVKHPELFEALGIAQPKGVLLYGPPGTGKTLLARAVAHHTDCTFIRVSGSELVQK
H. sapiens 2   155 EVIELPVKHPELFEALGIAQPKGVLLYGPPGTGKTLLARAVAHHTDCTFIRVSGSELVQK
D. melanogaster162 EVIELPVKHPELFDALGIAQPKGVLLYGPPGTGKTLLARAVAHHTECTFIRVSGSELVQK
A. thaliana a  175 EVIELPIKHPELFESLGIAQPKGVLLYGPPGTGKTLLARAVAHHTDCTFIRVSGSELVQK
A. thaliana b  175 EVIELPIKHPELFESLGIAQPKGVLLYGPPGTGKTLLARAVAHHTDCTFIRVSGSELVQK
N. tabacum     173 EVIELPIKHPELFESLGIAQPKGVLLYGPPGTGKTLLARAVAHHTDCTFIRVSGSELVQK
C. annuum      173 EVIELPIKHPELFESLGIAQPKGVLLYGPPGTGKTLLARAVAHHTDCTFIRVSGSELVQK
O. sativa      180 EVIELPIKHPELFESLGIAQPKGVLLYGPPGTGKTLLARAVAHHTDCTFIRVSGSELVQK
S. cerevisiae  162 EVIELPVKHPELFESLGIAQPKGVILYGPPGTGKTLLARAVAHHTDCKFIRVSGAELVQK
```

```
M. musculus     223 FIGEGARMVRELFVMAREHAPSIIFMDEIDSIGSSRLEGGSG-GDSEVQRTMLELLNQLD
R. norvegicus   223 FIGEGARMVRELFVMAREHAPSIIFMDEIDSIGSSRLEGGSG-GDSEVQRTMLELLNQLD
H. sapiens 1    223 FIGEGARMVRELFVMAREHAPSIIFMDEIDSIGSSRLEGGSG-GDSEVQRTMLELLNQLD
H. sapiens 2    215 FIGEGARMVRELFVMAREHAPSIIFMDEIDSIGSSRLEGGSG-GDSEVQRTMLELLNQLD
D. melanogaster222 FIGEGSRMVRELFVMAREHAPSIIFMDEIDSIGSSRIESGSG-GDSEVQRTMLELLNQLD
A. thaliana a   235 YIGEGSRMVRELFVMAREHAPSIIFMDEIDSIGSARMESGSGNGDSEVQRTMLELLNQLD
A. thaliana b   235 YIGEGSRMVRELFVMAREHAPSIIFMDEIDSIGSARMESGSGNGDSEVQRTMLELLNQLD
N. tabacum      233 YIGEGSRMVRELFVMAREHAPSIIFMDEIDSIGSARMESGSGNGDSEVQRTMLELLNQLD
C. annuum       233 YIGEGSRMVRELFVMAREHAPSIIFMDEIDSIGSARMESGSGNGDSEVQRTMLELLNQLD
O. sativa       240 YIGEGSRMVRELFVMAREHAPSIIFMDEIDSIGSARMESGTGNGDSEVQRTMLELLNQLD
S. cerevisiae   222 YIGEGSRMVRELFVMAREHAPSIIFMDEIDSIGSTRVEGSGG-GDSEVQRTMLELLNQLD

M. musculus     282 GFEATKNIKVIMATNRIDILDSALLRPGRIDRKIEFPPPNEEARLDILKIHSRKMNLTRG
R. norvegicus   282 GFEATKNIKVIMATNRIDILDSALLRPGRIDRKIEFPPPNEEARLDILKIHSRKMNLTRG
H. sapiens 1    282 GFEATKNIKVIMATNRIDILDSALLRPGRIDRKIEFPPPNEEARLDILKIHSRKMNLTRG
H. sapiens 2    274 GFEATKNIKVIMATNRIDILDSALLRPGRIDRKIEFPPPNEEARLDILKIHSRKMNLTRG
D. melanogaster281 GFEATKNIKVIMATNRIDILDPALLRPGRIDRKIEFPPPNEEARLDILKIHSRKMNLTRG
A. thaliana a   295 GFEASNKIKVLMATNRIDILDQALLRPGRIDRKIEFPNPNEESRFDILKIHSRKMNLMRG
A. thaliana b   295 GFEASNKIKVLMATNRIDILDQALLRPGRIDRKIEFPNPNEESRFDILKIHSRKMNLMRG
N. tabacum      293 GFEASNKIKVLMATNRIDILDQALLRPGRIDRKIEFPNPNEESRLDILKIHSRKMNLMRG
C. annuum       293 GFEASNKIKVLMATNRIDILDQALLRPGRIDRKIEFPNPNEESRFDILKIHSRKMNLMRG
O. sativa       300 GFEASNKIKVLMATNRIDILDQALLRPGRIDRKIEFPNPNEDSRFDILKIHSRKMNLMRG
S. cerevisiae   281 GFETSKNIKIIMATNRLDILDPALLRPGRIDRKIEFPPPSVAARAEILRIHSRKMNLTRG

M. musculus     342 INLRKIAELMPGASGAEVKGVCTEAGMYALRERRVHVTQEDFEMAVAKVMQKDSEKNMSI
R. norvegicus   342 INLRKIAELMPGASGAEVKGVCTEAGMYALRERRVHVTQEDFEMAVAKVMQKDSEKNMSI
H. sapiens 1    342 INLRKIAELMPGASGAEVKGVCTEAGMYALRERRVHVTQEDFEMAVAKVMQKDSEKNMSI
H. sapiens 2    334 INLRKIAELMPGASGAEVKGVCTEAGMYALRERRVHVTQEDFEMAVAKVMQKDSEKNMSI
D. melanogaster341 INLRKIAELMPGASGAEVKGVCTEAGMYALRERRVHVTQEDFEMAVAKVMQKDSEKNMSI
A. thaliana a   355 IDLKKIAEKMNGASGAELKAVCTEAGMFALRERRVHVTQEDFEMAVAKVMKKDTEKNMSL
A. thaliana b   355 IDLKKIAEKMNGASGAELKAVCTEAGMFALRERRVHVTQEDFEMAVAKVMKKDTEKNMSL
N. tabacum      353 IDLKKIAEKMNGASGAELKAVCTEAGMFALRERRVHVTQEDFEMAVAKVMKKETEKNMSL
C. annuum       353 IDLKKIAEKMNGASGAELKAVCTEAGMFALRERRVHVTQEDFEMAVAKVMKKETEKNMSL
O. sativa       360 IDLKKIAEKMNGASGAELKAVCTEAGMFALRERRVHVTQEDFEMAVAKVMKKDTEKNMSL
S. cerevisiae   341 INLRKVAEKMNGCSGADVKGVCTEAGMYALRERRIHVTQEDFELAVGKVMNKNQETAISV

M. musculus     402 KKLWK
R. norvegicus   402 KKLWK
H. sapiens 1    402 KKLWK
H. sapiens 2    394 KKLWK
D. melanogaster401 KKLWK
A. thaliana a   415 RKLWK
A. thaliana b   415 RKLWK
N. tabacum      413 RKLWK
C. annuum       413 RKLWK
O. sativa       420 RKLWK
S. cerevisiae   401 AKLFK
```

B

	M. musculus	*R. norvegicus*	*H. sapiens 1*	*H. sapiens 2*	*D. melanogaster*	*A. thaliana a*	*A. thaliana b*	*N. tabacum*	*C. annuum*	*O. sativa*	*S. cerevisiae*
M. musculus		100	100	98	91	79	78	80	79	77	74
R. norvegicus			100	98	91	79	79	80	79	77	74
H. sapiens 1				98	91	79	79	80	79	77	74
H. sapiens 2					95	78	78	79	79	76	74
D. melanogaster						78	78	79	79	76	73
A. thaliana a							97	93	93	92	70
A. thaliana b								91	91	91	69
N. tabacum									98	91	70
C. annuum										91	70
O. sativa											68

Figure S1: Protein sequence alignment of RPT6 from different species. (A) The alignment was generated using CLUSTALW2 with default parameters and BoxShade 3.21. Positions of identical and similar sequences are boxed in black and grey, respectively. The following sequences were used to build the alignment: *Mus musculus* NP032976*; Rattus norvegicus* BAA22935; *Homo sapiens 1* NP_002796.4; *Homo sapiens 2* NP_001186092.1; *Drosophila melanogaster* NP_608447.1; *Arabidopsis thaliana* isoform a At5g19990; *Arabidopsis thaliana* isoform b At5g20000; *Nicotiana tabacum* JX965405; *Capsicum annuum* JX965404, *Oryza sativa* NP_001046248.1; *Saccharomyces cerevisiae* EHN02423.1 (B) Similarity matrix of RPT6 sequences from different species. Degree of similarity is given in percent.
