# Supplementary material for: The Xanthomonas campestris Type III Effector XopJ Targets the Host Cell Proteasome to Suppress Salicylic-Acid Mediated Plant Defence
Source: PLoS Pathog. 2013 Jun 13;9(6):e1003427. doi: 10.1371/journal.ppat.1003427 (PMC3681735; doi:10.1371/journal.ppat.1003427)
Supplement: Figure S2 — Control experiments for BiFC assays. YFP confocal microscopy images show tobacco leaf epidermal cells transiently expressing constructs encoding the fusion proteins indicated. Merge indicates an overlay of the YFP and chlorophyll autofluorescence images. Each image is the representative of at least two experiments. Bars = 10 µm if not otherwise indicated. (PDF) [file ppat.1003427.s002.pdf]

Figure S2

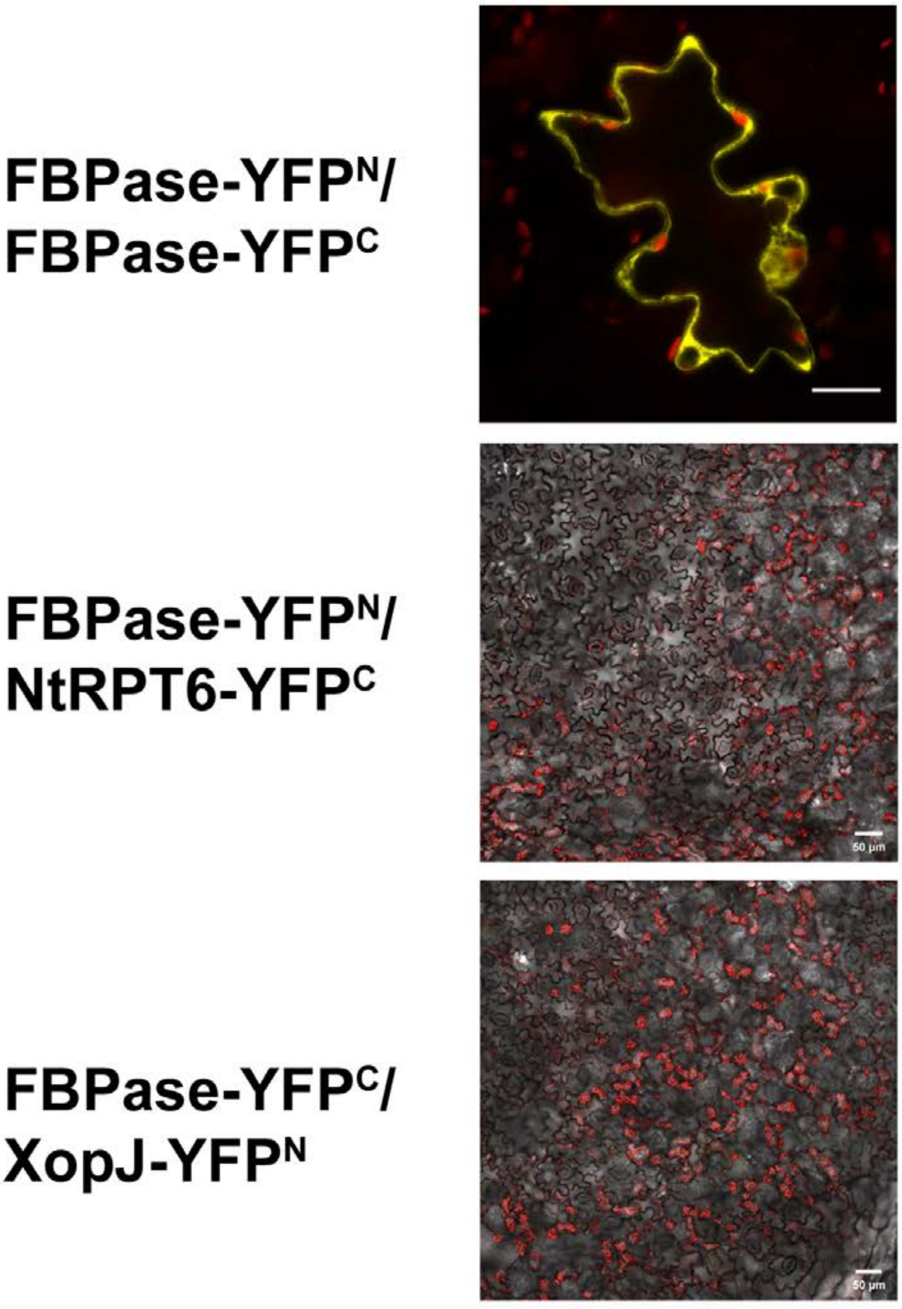

Figure S2: Control experiments for BiFC assays. YFP confocal microscopy images show a merge YFP- and chlorophyll auto-fluorescence of tobacco leaf epidermal cells transiently expressing constructs encoding the fusion proteins indicated. Each image is the representative of at least three experiments.
