# Supplementary material for: The Xanthomonas campestris Type III Effector XopJ Targets the Host Cell Proteasome to Suppress Salicylic-Acid Mediated Plant Defence
Source: PLoS Pathog. 2013 Jun 13;9(6):e1003427. doi: 10.1371/journal.ppat.1003427 (PMC3681735; doi:10.1371/journal.ppat.1003427)
Supplement: Figure S3 — Proteasome inhibition is specific for XopJ. (A) XopJ protein along with an empty vector (EV) control and XopB-myc were transiently expressed in leaves of N. benthamiana using Agro-infiltration. After 48 h, relative proteasome activity in total protein extracts was determined by monitoring the breakdown of the fluorogenic peptide Suc-LLVY-NH-AMC at 30°C in a fluorescence spectrophotometer. The empty vector (EV) control was set to 100%. Data represent the mean + SD (n = 3). (B) Immunodetection of transiently expressed XopJ and XopB in the same leaves that were used for proteasome activity measurements. After immunodetection of proteins the membrane was stained with amido black to control for equal protein loading. (PDF) [file ppat.1003427.s003.pdf]

Figure S3

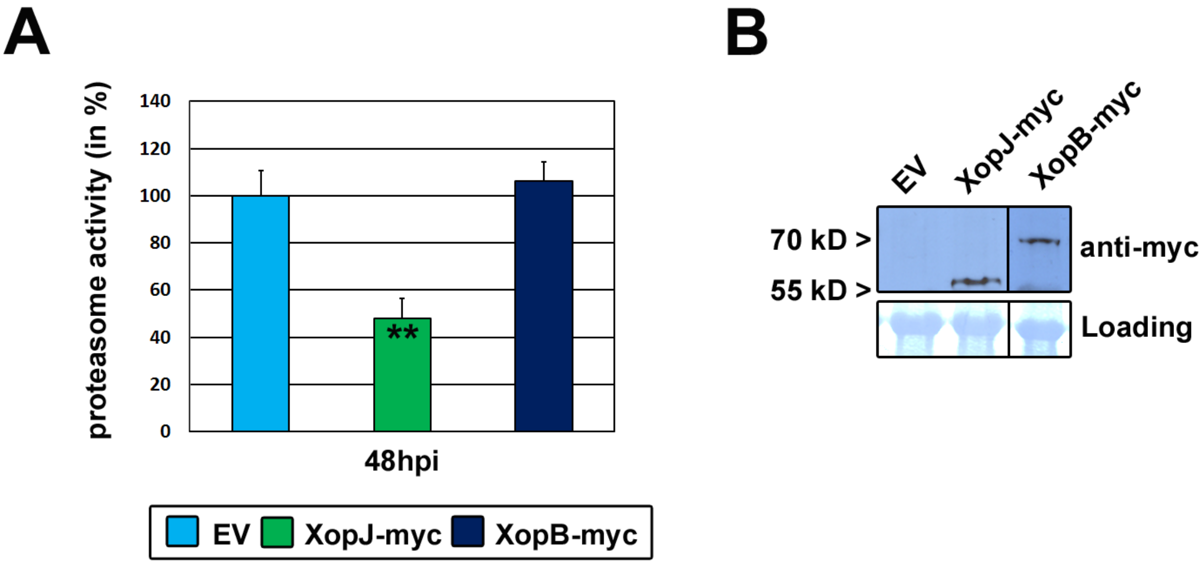

Figure S3: Proteasome inhibition is specific for XopJ. **(A)** XopJ protein along with an empty vector (EV) control and XopB-myc were transiently expressed in leaves of *N. benthamiana* using Agro-infiltration. After 48 h, relative proteasome activity in total protein extracts was determined by monitoring the breakdown of the fluorogenic peptide Suc-LLVY-NH-AMC at 30°C in a fluorescence spectrophotometer. The empty vector (EV) control was set to 100 %. Data represent the mean + SD (n = 3). **(B)** Immunodetection of transiently expressed XopJ and XopB in the same leaves that were used for proteasome activity measurements. After immunodetection of proteins the membrane was stained with amido black to control for equal protein loading.
