# Supplementary material for: The Xanthomonas campestris Type III Effector XopJ Targets the Host Cell Proteasome to Suppress Salicylic-Acid Mediated Plant Defence
Source: PLoS Pathog. 2013 Jun 13;9(6):e1003427. doi: 10.1371/journal.ppat.1003427 (PMC3681735; doi:10.1371/journal.ppat.1003427)
Supplement: Figure S4 — Phenotype of Xcv infected pepper leaves 5 dpi. Xcv (vector), Xcv ΔxopJ (vector), Xcv ΔxopJ (XopJ-HA), XopJ (G2A-HA) and (C235A-HA) were inoculated at a bacterial density of 2×10^8^ cfu ml−1 into leaves of pepper ECW plants. Pictures were taken at 5 dpi. (PDF) [file ppat.1003427.s004.pdf]

Figure S4

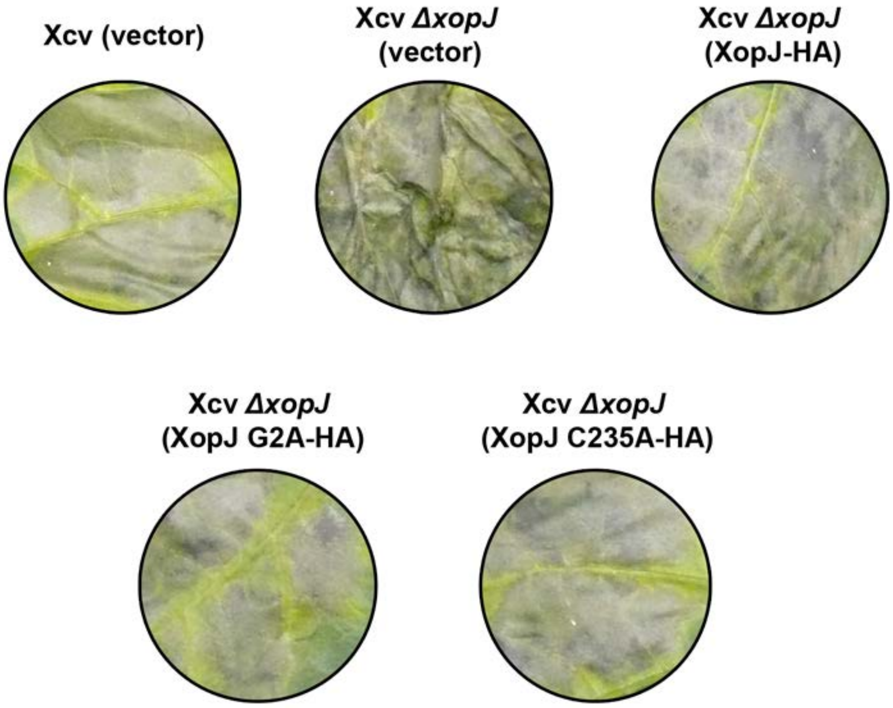

Figure S4: Phenotype of Xcv infected pepper leaves 5 dpi. Xcv (vector), Xcv *ΔxopJ* (vector), Xcv *ΔxopJ* (XopJ-HA), XopJ (G2A-HA) and (C235A-HA) were inoculated at a bacterial density of 2 x 10^8 cfu ml^{-1} into leaves of pepper ECW plants. Pictures were taken at 5 dpi.
