# Supplementary material for: The Xanthomonas campestris Type III Effector XopJ Targets the Host Cell Proteasome to Suppress Salicylic-Acid Mediated Plant Defence
Source: PLoS Pathog. 2013 Jun 13;9(6):e1003427. doi: 10.1371/journal.ppat.1003427 (PMC3681735; doi:10.1371/journal.ppat.1003427)
Supplement: Figure S5 — XopJ delivered by a complemented Xcv ΔxopJ (XopJ-HA) is able to suppress the phenotype of the Xcv Δ xopJ strain in a mixed inoculum experiment. Leaves of pepper ECW plants were first inoculated with Xcv ΔxopJ (XopJ-HA) at a density of 2×10^8^ cfu ml−1. After 3 h the same leaf region was inoculated with Xcv ΔxopJ bacteria of the same density. The phenotype of the infected leaf was recorded 3 dpi. (PDF) [file ppat.1003427.s005.pdf]

Figure S5

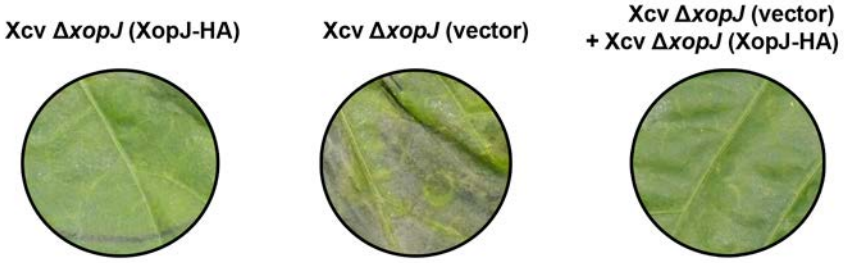

Figure S5: XopJ delivered by a complemented Xcv *ΔxopJ* (XopJ-HA) is able to suppress the phenotype of the Xcv *ΔxopJ* strain in a mixed inoculum experiment. Leaves of pepper ECW plants were first inoculated with Xcv Δ*xopJ* (XopJ-HA) at a density of 2 x 10^8 cfu ml^{-1}. After 3h the same leaf region was inoculated with Xcv Δ*xopJ* bacteria of the same density. The phenotype of the infected leaf was recorded 3 dpi.
