# Supplementary material for: The Xanthomonas campestris Type III Effector XopJ Targets the Host Cell Proteasome to Suppress Salicylic-Acid Mediated Plant Defence
Source: PLoS Pathog. 2013 Jun 13;9(6):e1003427. doi: 10.1371/journal.ppat.1003427 (PMC3681735; doi:10.1371/journal.ppat.1003427)
Supplement: Figure S6 — SA treatment induces tissue necrosis in Xcv wild type infected pepper leaves. Xcv wild type and Xcv ΔxopJ were inoculated at a bacterial density of 2×10^8^ cfu ml−1 into leaves of pepper ECW plants. 2 dpi Xcv wild type infected leaves were sprayed with 5 mM SA (middle) and the phenotype development was documented 3 dpi. Xcv wild type infected leaf sprayed with water (left) served as a control. For comparison phenotype development 3 dpi of aXcv ΔxopJ infected leaf is shown (right). (PDF) [file ppat.1003427.s006.pdf]

Figure S6

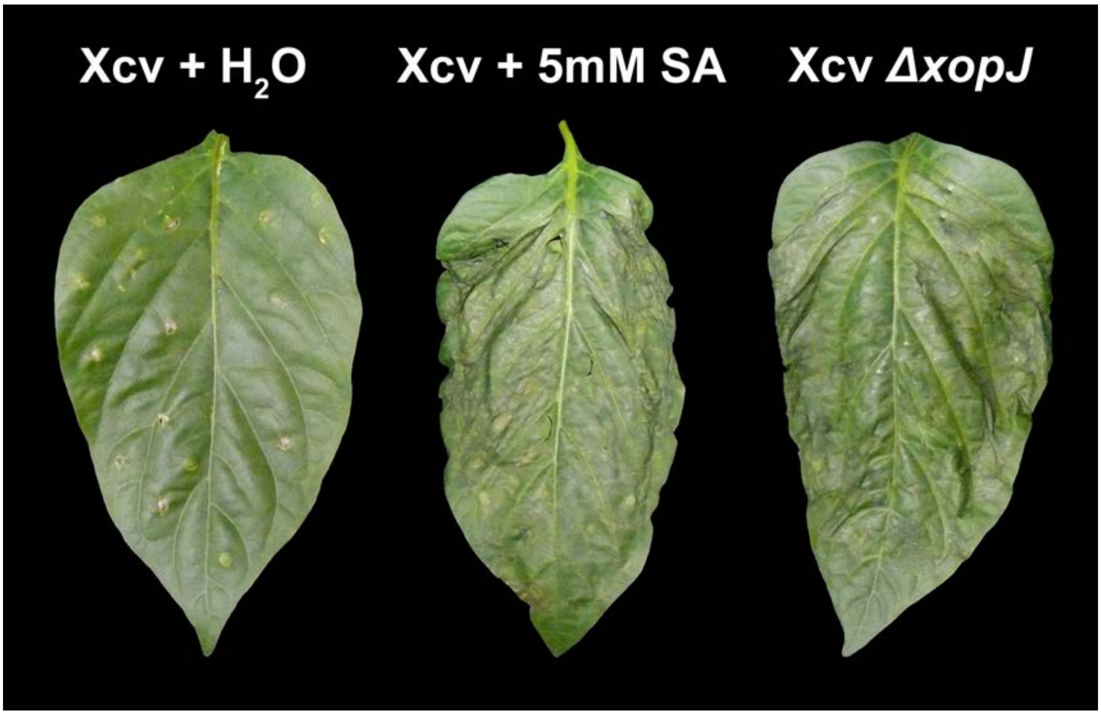

Figure S6: SA treatment induces tissue necrosis in Xcv wild type infected pepper leaves. Xcv wild type and Xcv *ΔxopJ* were inoculated at a bacterial density of 2 x 10^8 cfu ml^{-1} into leaves of pepper ECW plants. 2 dpi Xcv wild type infected leaves were sprayed with 5 mM SA (middle) and the phenotype development was documented 3 dpi. Xcv wild type infected leaf sprayed with water (left) served as a control. For comparison phenotype development 3 dpi of aXcv *ΔxopJ* infected leaf is shown (right).
