# Supplementary material for: The Xanthomonas campestris Type III Effector XopJ Targets the Host Cell Proteasome to Suppress Salicylic-Acid Mediated Plant Defence
Source: PLoS Pathog. 2013 Jun 13;9(6):e1003427. doi: 10.1371/journal.ppat.1003427 (PMC3681735; doi:10.1371/journal.ppat.1003427)
Supplement: Figure S7 — RPT6 gene expression during compatible Xcv-pepper interaction. Total RNA was isolated from pepper leaves infiltrated with 2×10^8^ cfu/mL of Xcv, Xcv ΔxopJ and 1 mM MgCl2. Quantitative real-time RT-PCR was performed for CaRPT6 three dpi. Actin expression was used to normalize the expression value in each sample, and relative expression values were determined against the average value of the sample infected with 1 mM MgCl2. Leaf material from 4 independent pepper plants was pooled and analyzed in triplicates. Data represent the mean SD. Significant differences were calculated using Student's t-test and are indicated by: *, P<0.05; **, P<0.01. (PDF) [file ppat.1003427.s007.pdf]

Figure S7

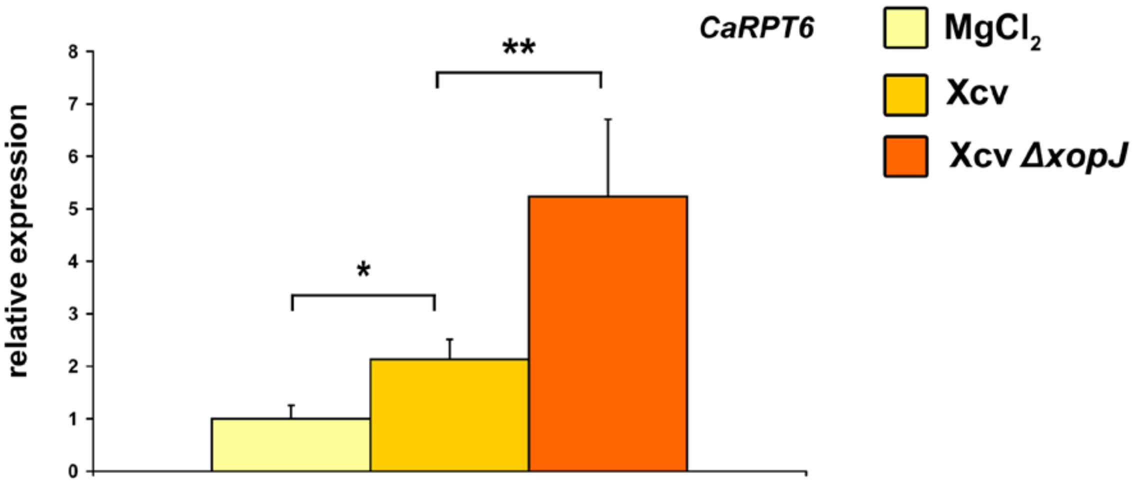

Figure S7: RPT6 gene expression during compatible Xcv-pepper interaction

Total RNA was isolated from pepper leaves infiltrated with 2 x 10^8 cfu/mL of Xcv, Xcv *ΔxopJ* and 1mM $MgCl_2$. Quantitative real-time RT-PCR was performed for *CaRPT6* three dpi. *Actin* expression was used to normalize the expression value in each sample, and relative expression values were determined against the average value of the sample infected with 1mM $MgCl_2$. Leaf material from 4 independent pepper plants was pooled and analyzed in triplicates. Data represent the mean SD. Significant differences were calculated using Student's t-test and are indicated by: *, $P < 0.05$; **, $P < 0.01$
