# Supplementary material for: The Xanthomonas campestris Type III Effector XopJ Targets the Host Cell Proteasome to Suppress Salicylic-Acid Mediated Plant Defence
Source: PLoS Pathog. 2013 Jun 13;9(6):e1003427. doi: 10.1371/journal.ppat.1003427 (PMC3681735; doi:10.1371/journal.ppat.1003427)
Supplement: Figure S8 — XopJ does not inhibit flg22-triggered MAPK signalling in Arabidopsis. (A) flg22 mediated MAPK activation un-induced alc-XopJ plant in comparison to those induced with ethanol. Leaves were treated with 1 µM flg22, and MAPK activation was analyzed at the indicated time points. Upper panel: protein extracts (15 µg per lane) were subjected to western-blot analysis using the phospho-p44/p42 MAPK antibody. Arrowheads indicate the positions of MPK3 and MPK6. Lower panel: equal protein level of MPK3 and MPK6 in each lane was verified using an anti-MPK6 antibody. (B) Proteasome activity before and after treatment of alc-XopJ plants with 1% ethanol. (PDF) [file ppat.1003427.s008.pdf]

Figure S8

A

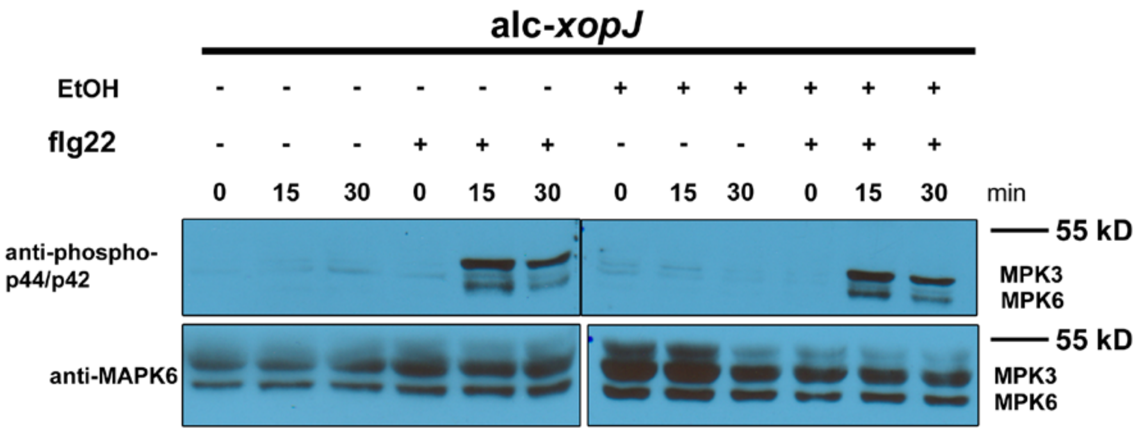

B

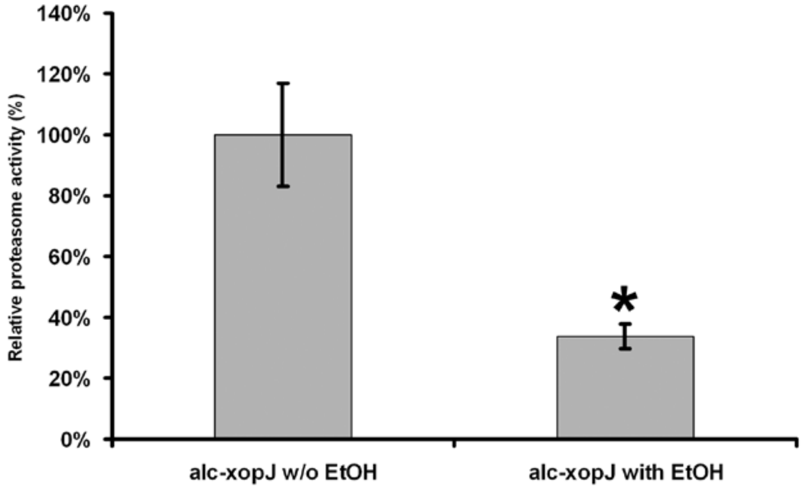

Figure S8: XopJ does not inhibit flg22-triggered MAPK signalling in Arabidopsis. (A) flg22 mediated MAPK activation un-induced alc-XopJ plant in comparison to those induced with ethanol. Leaves were treated with 1 µM flg22, and MAPK activation was analyzed at the indicated time points.Upper panel: protein extracts (15 μg per lane) were subjected to western-blot analysis using the phospho-p44/p42 MAPK antibody. Arrowheads indicate the positions of MPK3 and MPK6. Lower panel: equal protein level of MPK3 and MPK6 in each lane was verified using an anti-MPK6 antibody. (B) Proteasome activity before and after treatment of alc-XopJ plants with 1% ethanol. Data represent the mean SD. Significant differences were calculated using Student’s t-test and are indicated by: *, P < 0.05.
