# Supplementary material for: The Xanthomonas campestris Type III Effector XopJ Targets the Host Cell Proteasome to Suppress Salicylic-Acid Mediated Plant Defence
Source: PLoS Pathog. 2013 Jun 13;9(6):e1003427. doi: 10.1371/journal.ppat.1003427 (PMC3681735; doi:10.1371/journal.ppat.1003427)
Supplement: Figure S9 — Virus-induced gene silencing of RPT6 in ECW pepper plants. (A) qRT-PCR analysis of RPT6 mRNA level in RPT6 silenced pepper plants. The log2 value is given, where −3.32 corresponds to a 10-fold down-regulation in the VIGS – RPT6 plants compared with the control (B) Phenotype of RPT6 - VIGS plants in comparison to the pTRV2-GFPsil control. Picture was taken 21 dpi. (PDF) [file ppat.1003427.s009.pdf]

Figure S9

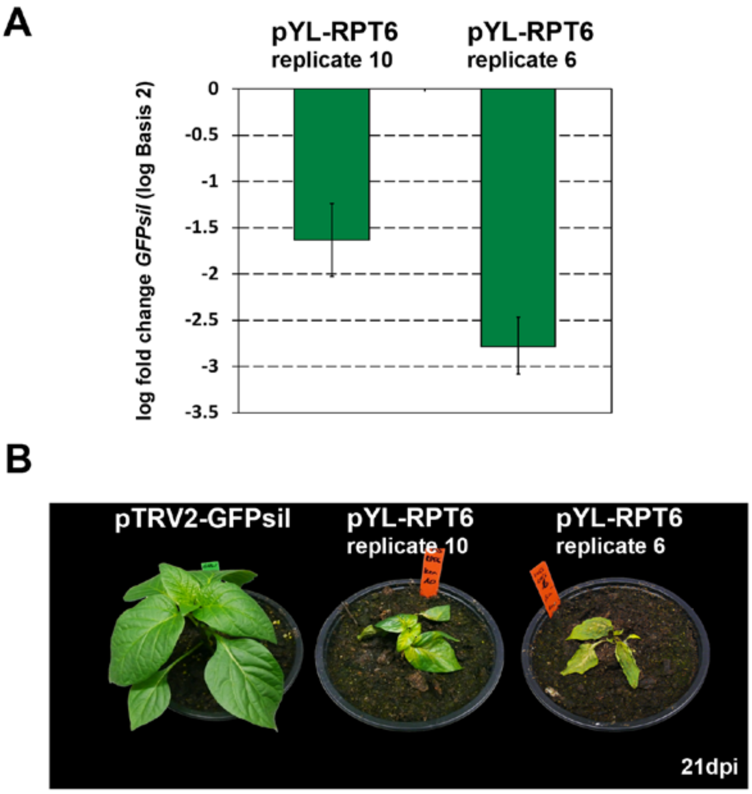

Figure S9: Virus-induced gene silencing of RPT6 in ECW pepper plants. (A) qRT-PCR analysis of RPT6 mRNA level in RPT6 silenced pepper plants. The $\log_2$ value is given, where -3.32 corresponds to a 10-fold down-regulation in the VIGS – RPT6 plants compared with the control (B) Phenotype of RPT6 - VIGS plants in comparison to the pTRV2-GFPsil control. Picture was taken 21 dpi.
