# Supplementary material for: The Xanthomonas campestris Type III Effector XopJ Targets the Host Cell Proteasome to Suppress Salicylic-Acid Mediated Plant Defence
Source: PLoS Pathog. 2013 Jun 13;9(6):e1003427. doi: 10.1371/journal.ppat.1003427 (PMC3681735; doi:10.1371/journal.ppat.1003427)
Supplement: Table S1 — Oligonucleotides used in this study. (PDF) [file ppat.1003427.s010.pdf]

**Supplemental Table 1: Primers used in this study.**		
Nucleotide sequence of gene-specific primers for interaction tests		
XopJ G2A	Fw	5’-GGATCCTGGCTCTATGCGTTTCAAAGCCG-3’
	Rv	5’-GTCGACCTATGACTGGCGATCAGAGAT-3’
XopJ C235A	Fw	5’-CAGAAGTCTGCAGCGGACGCCCTGATGTTCGATCTGCAT-3’
	Rv	5’-ATGCAGATCGAACATCAGGGCGTCCGCTGCAGACTTCTG-3’
*Nt*RPT6	Fw	5’-CACCGAGGAAATGGCGTCAGCTGATG-3’
	Rv	5’-CTTCCACAGCTTTCGCAGTGAC-3’
*At*RPT6a	Fw	5’-GGGATCCGTAGTGCGGCCGTAGGAGTAG-3’
	Rv	5’-GTCGACCTACTTCCACAGCTTACGCAG-3’
*At*RPT6b	Fw	5’-GGGATCCGTATGGCGGCCGTAGGAGTGGAGG-3’
	Rv	5’-GTCGACCTACTTCCAGAGCTTACGCAGAGAC-3’
*Sc*RPT6	Fw	5’-GGGATCCGTATGACAGCTGCTGTAACATCCTCC-3’
	Rv	5’-GTCGACTCACTTGAACAGCTTGGCGACAG-3’
Nucleotide sequence of gene-specific primers for the construction of the Xcv *ΔxopJ* mutant		
ΔxopJ P1	Fw	5’-GGATCCGGTGAGCCAGCGCGAATC-3’
	Rv	5’-GTAACCTCGATCTACGCGCAACGCTGGGTCAGTGGC-3’
ΔxopJ P2	Fw	5’-GCCACTGACCCAGCGTTGCGCGTAGATCGAGGTTAC-3
	Rv	5’-GTCGACCGTCGTCGCGGCAAAGAG-3’
Nucleotide sequence of gene-specific primers for construction of C-terminal fusions		
XopJ	Fw	5’-CACCGTAACAATGGGTCTATGCGTTTCAAAG -3’
	Rv	5’- GGATCCTGACTGGCGATCAGAGATAGC-3’
XopJ C235A	Fw	5’-CAGAAGTCTGCAGCGGACGCCCTGATGTTCGATCTGCAT-3’
	Rv	5’-ATGCAGATCGAACATCAGGGCGTCCGCTGCAGACTTCTG-3’
XopJ G2A	Fw	5'-CACCGTAACAATGGCTCTATGCGTTTCAAAG-3'
	Rv	5’-GGATCCTGACTGGCGATCAGAGATAGC-3’
*Nt*RPT6	Fw	5’-CACCGAGGAAATGGCGTCAGCTGATG-3’
	Rv	5'-CTTCCACAGCTTTCGCAGTGAC-3'
Nucleotide sequence of gene-specific primers for quantitative real-time RT-PCR		
CaRPT6	Fw	5’-TGCAGCATATCCACGATCTC-3’
	Rv	5’-CAGGCTCCTGAAGCAACTGT-3’
NbRPT6	Fw	5’-AGGGAGTGGCAATGGTGATA-3’
	Rv	5’-AATCAATCCCTCGCATCAAG-3’
CaBPR1	Fw	5’-CAGGATGCAACACTCTGGTGG-3’
	Rv	5’-ATCAAAGGCCGGTTGGTC-3’
CaPR-Q	Fw	5’-AGGGCTTCTACACTTACGATGC-3’
	Rv	5’-TCCTTCTTACGGGCACTATCAT-3’
CaSAR82A	Fw	5’-CAGATTGTTGCCAGGGAGAT-3’
	Rv	5’-ACAACGGCCATGACAAGTTT-3’
CaSENU4	Fw	5’-GGCATCTCGAGCACAAAACT-3’
	Rv	5’-GCGCCAGACCACTTGAGTAT-3’
CaSGR	Fw	5’-CCAGGAAAGTTGCCAAGAAC-3’
	Rv	5’-GCCTCCACTAATGTGGCAAT-3’
CaCab-1b	Fw	5’-TCAAGTTTGGTGAGGCTGTG-3’
	Rv	5’-GGGATAGAGTGGGTCGACAA-3’
Actin	Fw	5’-GCCAACAGAGAGAAGATGACCCAGA-3’
	Rv	5’-ACACCATCACCAGAGTCCAACACAAT-3’
